# Alzheimer's Disease: Mechanisms and Therapeutic Advances

**DOI:** 10.1002/mco2.71093

**Published:** 2026-11-03

**Authors:** Gaoshuang Fu, Mingmin Pan, Qingling Sun, Guangxin Yue, Tong Lei

**Affiliations:** ^1^ Institute of Basic Theory For Chinese Medicine China Academy of Chinese Medical Sciences Beijing China

**Keywords:** Alzheimer's disease, cognitive impairment, drug delivery, inflammation, mental symptom, natural products

## Abstract

Alzheimer's disease (AD) is a devastating neurodegenerative condition accompanied by widespread central nervous system damage and complex networked pathogenesis spanning molecular, cellular, and systemic dimensions. Existing studies tend to focus on individual pathological cascades or monotherapeutic approaches, while overlooking the intrinsic crosstalk among protein pathology, neuroinflammation, metabolic dysfunction, and gut–brain axis imbalance, with insufficient systematic integration of nonpharmacological strategies and multifunctional nanoplatforms. Driven by intertwined molecular aberrations, glial dysregulation and systemic metabolic disorders, AD presents profound pathological complexity. Correspondingly, multitarget pharmacotherapy integrated with hierarchical nonpharmacological and nanotherapeutic interventions has achieved substantial progress, exerting synergistic effects on alleviating AD pathological cascades and optimizing overall therapeutic regimens. We comprehensively review current evidence to delineate the mechanistic landscape of AD and update therapeutic advances, including pharmacological agents, multimodal nonpharmacological interventions, and brain‐targeted nanosystems for blood–brain barrier penetration, protein modulation, microenvironment remodeling, and gene editing delivery. This study underscores major research limitations and translational obstacles in therapeutic efficacy, biosafety, and nanomedicine applications. This work emphasizes biomarker‐based early stratification and staged combined interventions, providing novel integrated insights into AD pathogenesis and prospective targeted therapeutic development.

## Graphical abstract

Alzheimer's disease involves Aβ/tau aggregation, neuroinflammation, BBB leakage, gut–brain dysfunction, and mitochondrial damage, causing neuronal loss. Nanodelivery enables combined small‐molecule and targeted therapies. Disease‐modifying biologics/chemicals, including anti‐Aβ antibodies, raise safety and access concerns. Exercise, cognitive training, and gut regulation offer safe adjuvant and preventive strategies.

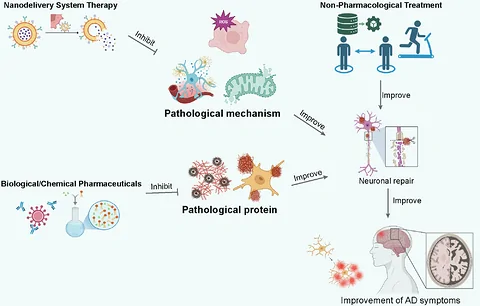

## 1 Introduction

Alzheimer's disease (AD), a progressive neurodegenerative disorder, represents the most prevalent form of dementia, and its rising incidence poses a severe challenge to the global public health system [1]. Since first reported a case characterized by progressive memory impairment, neuropsychiatric abnormalities, and distinctive neuropathological alterations in 1906, research on AD has evolved for over a century. Early studies primarily focused on senile plaques and neurofibrillary tangles (NFTs), and thereafter a classic pathological framework centered on amyloid‐β (Aβ) deposition and abnormal hyperphosphorylation of tau protein was gradually established. Among relevant theories, the amyloid cascade hypothesis has long dominated the interpretation of AD pathogenesis and the direction of drug development. This hypothesis proposes that abnormal production, aggregation, and impaired clearance of Aβ act as upstream events that trigger tau pathology, synaptic damage, neuronal loss, and cognitive decline [1, 2, 3].

Nevertheless, accumulating findings from basic research and clinical trials in recent years have continuously indicated that AD cannot be fully explained by the deposition of a single pathological protein. Although Aβ and tau remain the core targets for AD diagnosis and therapeutic research, growing evidence demonstrates that AD is a complex systemic disease involving the impaired protein homeostasis, neuroimmune dysfunction, blood–brain barrier (BBB) impairment, and multiple other pathological processes [4, 5]. In terms of treatment, symptomatic medications such as cholinesterase inhibitors (ChEIs) and N‐methyl‐D‐aspartate (NMDA) receptor antagonists have long been the mainstay of AD management. These drugs can ameliorate cognitive deficits, behavioral disturbances, and activities of daily living (ADL) to a certain extent, yet they fail to halt the pathological progression of the disease [6]. In recent years, the approval of anti‐Aβ monoclonal antibodies has advanced AD therapy into the era of disease‐modifying treatment. The clinical application of agents including lecanemab and donanemab has verified that pathological protein clearance in patients with early AD may delay cognitive and functional deterioration [7, 8, 9, 10]. Furthermore, emerging strategies targeting tau protein, active immunization vaccines, immune regulation, repurposing of antidiabetic drugs, as well as nonpharmacological interventions have broadened the research landscape for AD treatment [11, 12, 13, 14, 15]. However, current therapeutic approaches still confront multiple challenges, including limited efficacy, potential safety risks, stringent patient screening criteria, high treatment costs, and unclear long‐term clinical benefits (Table 1).

**TABLE 1:** New advances in AD pharmacotherapy.

Drug class	Drug name	Participants	Intervention details	Comparison	Prespecified endpoints	Observed results	Adverse reactions	Clinical trial phase	References
Aβ monoclonal antibody	Lecanemab	MCI and mild AD	IV 10 mg/kg Q2W for 18 months	Placebo	Primary: CDR‐SB at 18 months; secondary: amyloid PET, ADAS‐Cog14, ADCOMS, ADCS‐ADL‐MCI	Met primary endpoint; CDR‐SB +1.21 vs. +1.66, *p* < 0.001; amyloid PET Δ ≈ −59.1 centiloids; all key secondary endpoints significant	Infusion‐related reactions 26.4%: ARIA‐E 12.6%: ARIA‐H 17.3%; elevated d‐dimer; severe urinary retention	Phase 3 completed	[7, 117, 118]
Aβ monoclonal antibody	Donanemab	Early AD, amyloid+/tau+	IV 700 mg Q4W for the first 3 doses, followed by 1400 mg Q4W, for up to 18 months	Placebo	Primary: iADRS change at 76 weeks in low/medium‐tau and combined populations; secondary: CDR‐SB, ADAS‐Cog13, ADCS‐iADL, amyloid PET, and so on	Met primary endpoint; low/medium tau: iADRS decline slowed 35.1%; combined population ≈22.3%; CDR‐SB slowing ≈36.0 and 28.9%, respectively	ARIA‐E 24.0%; ARIA‐H 31.4%; infusion reaction 8.7%; serious ARIA including deaths occurred	Phase 3 completed	[9]
Aβ monoclonal antibody	Donanemab	Early AD and amyloid+	IV donanemab Q4W: 700 mg for the first 3 doses, followed by 1400 mg Q4W, for up to 18 months	Aducanumab	Primary: proportion of participants achieving amyloid plaque clearance (<24.1 centiloids) at Month 6; secondary: amyloid plaque burden and clearance at Months 12 and 18, time to amyloid clearance, plasma biomarkers (p‐tau217, p‐tau181, GFAP, and NfL), and safety/ARIA	Met primary endpoint; amyloid clearance at Month 6 was 37.9 vs. 1.6% (*p* < 0.001); clearance rates were 70.0 vs. 24.6% at Month 12 and 76.8 vs. 43.1% at Month 18; median time to amyloid clearance was 359 vs. 568 days (*p* < 0.001); at Month 18, plasma p‐tau217 decreased by 33.2 vs. 25.7% from baseline.	ARIA‐E: 23.9 vs. 34.8%; any TEAE: 83.1 vs. 87.0%; treatment‐related TEAEs: 36.6 vs. 46.4%; serious AEs: 18.3 vs. 11.6%; no deaths occurred during the study	Phase 3 completed	[10]
Aβ monoclonal antibody	ALZ‐801	APOE4 carriers with early AD	Oral 265 mg QD for 2 weeks, then 265 mg BID for 104 weeks	No concurrent control	Changes in AD biomarkers, hippocampal volume, cognition, safety, and pharmacokinetics	Reduction in plasma p‐tau181 and inhibition of hippocampal atrophy; cognition remained relatively stable; pharmacokinetics showed low intersubject variability	Mild nausea; no ARIA observed	Phase 2 completed	[119, 120]
Tau monoclonal antibody	BMS‐986446	Early AD	IV 1400 or 4200 mg Q4W for 76 weeks	Placebo	Efficacy/safety; clinical measures plus tau PET, MRI, and fluid biomarkers	No definitive efficacy readout yet	Not available	Phase 2 ongoing	[121]
Tau monoclonal antibody	Bepranemab	MCI and mild AD	IV for 80 weeks	Placebo	Clinical efficacy including CDR‐SB; tau‐PET and other biomarkers	Failed to meet primary CDR‐SB endpoint; reduced CDR‐SB and ADAS‐Cog and slowed tau deposition in patients with low baseline tau and non‐ApoE4 carriers.	Overall safety, treatment‐related events monitored	Phase 2 completed	[122]
Tau monoclonal antibody	E2814	MCI and mild AD	IV for 24 weeks, then combined with E2814 or placebo for 4 years	Placebo	Symptomatic cohort: tau‐PET spread; asymptomatic cohort: CSF p‐tau217/total tau; CDR‐SB, biomarkers, and so on	Not available	Not available	Phase 2/3 ongoing	[123, 124]
Tau‐targeting antisense oligonucleotide	Diranersen	MCI and mild AD	Intrathecal injection 60 mg Q24W, 115 mg Q24W or Q12W for 76 weeks	Placebo	Primary: dose–response relationship in CDR‐SB at Week 76; secondary: CDR‐SB, ADAS‐Cog13, MMSE, modified iADRS, ADCOMS, and so on; CSF tau/tau‐PET	Primary dose‐response endpoint not met. Nevertheless 60 mg showed strongest signal: CDR‐SB 26%, ADAS‐Cog13 42%, MMSE 50% slowing; CSF total tau reduced 50–65%; tau PET decreased	Generally well tolerated; procedural pain, postlumbar‐puncture syndrome, confusional state prominent	Phase 2 completed	[125]
Aβ vaccine	CAD106	Mild AD	IM 150 or 450 µg up to 7 times within 60 weeks	Placebo	Time to MCI/dementia due to AD; cognition/function, biomarkers, safety/immunogenicity	Study terminated early; therefore no definitive demonstration of prevention of cognitive decline	Asymptomatic ARIA in strong responders; immunogenicity/safety assessed; program terminated	Phase 2b completed	[12]
Aβ vaccine	UB‐311	Mild AD	IM up to 7 times within 78 weeks	Placebo	Primary: safety, tolerability, immunogenicity; secondary: exploratory cognitive efficacy	Safe and immunogenic; 97% antibody response, 93% maintained at study end; small trial not powered to establish clinical efficacy	Injection‐site pain 16%; ARIA‐H/microhemorrhage/hemosiderin deposits 14%; diarrhea 12%	Phase 2a completed	[126, 127]
Tau vaccine	AADvac1	Mild AD	IM 6 doses Q4W, followed by 5 booster doses Q14W for 24 months	Placebo	Primary: safety/tolerability; secondary: immunogenicity, cognition/function, biomarkers	Safe and highly immunogenic; no significant clinical benefit; reduced accumulation rate of NfL and GFAP in plasma p‐tau217‐positive participants	Injection site reaction	Phase 2 completed	[128, 129]
Tau vaccine	ACI‐35.030	Early AD	300, 900, 1800 µg IM, 4 injections within 48 weeks	Placebo	Primary: safety, tolerability, immunogenicity. Secondary: biomarkers exploratory	Sustained increase in immunogenicity after booster; reduced plasma p‐Tau217 and brain‐derived Tau	Injection site reaction, headache	Phase 1b/2a completed; Phase 2b ongoing	[130]
Tau vaccine	JACI‐35.054	Early AD	15 or 60 µg IM, 4 injections within 48 weeks	Placebo	Safety, tolerability, immunogenicity	Strong but more heterogeneous anti‐p‐Tau response; required multiple administrations for consistent titers	Injection site reaction, headache	Phase 1b/2a completed	[130]
Tyrosine kinase inhibitor	Masitinib	Mild‐to‐moderate AD	Oral administration: 4.5 mg/kg/day for 12 weeks, followed by 6.0 mg/kg/day until Week 24	Placebo	Coprimary: ADAS‐Cog and ADCS‐ADL change at Week 24	4.5 mg/kg: ADAS‐Cog difference −2.15, *p* < 0.001; ADCS‐ADL difference +1.82, *p* = 0.038	Neutropenia, pneumonia, hypoalbuminemia, and Stevens–Johnson syndrome	Phase 3 completed	[131]
TREM2 agonist	AL002	Early AD subjects	IV; dose‐ranging	Placebo	Primary: CDR‐SB; secondary: clinical/function, microglial, biomarkers, and amyloid PET	Primary endpoint not met; no clinical/functional secondary endpoint; target engagement and microglial activation occurred, but no significant favorable AD fluid biomarker or amyloid PET effect	ARIA and injection site reaction	Phase 2 completed	[132]
Treg immune reconstitution	IL‐2	Mild to moderate AD	Subcutaneous administration (10^6^ IU/day) for 5 days, administered every 4 weeks or every 2 weeks for 21 weeks	Placebo	Safety/tolerability plus immune/clinical and biomarker	No definitive efficacy results yet	Not available	Phase 2 ongoing	[133]
GLP‐1RA	Liraglutide	Mild‐to‐moderate AD	Subcutaneous titrated to 1.8 mg daily for 52 weeks	Placebo	Primary: change in cerebral glucose metabolic rate; secondary: cognition, MRI, safety/tolerability	Primary endpoint not met; secondary analyses generated signals but do not overturn primary result.	Gastrointestinal symptoms and weight effects relevant	Phase 2b completed	[14]
GLP‐1RA	Semaglutide	MCI and mild AD	Oral flexible titration to 14 mg/d up to 156 weeks	Placebo	Primary: CDR‐SB change at Week 104; secondary: ADCS‐ADL‐MCI; progression to CDR global ≥1, and so on	Both Phase 3 trials negative; some biomarkers changed but no clinical benefit	Weight loss 36.5%, decreased appetite 33.1%, nausea 24.3%	Phase 3 completed and program discontinued	[134]
Intranasal insulin	Humulin‐RU‐100	MCI and mild AD	Intranasal administration 40 IU daily for 12 months	Placebo	Primary: ADAS‐Cog12 change at 12 month; secondary: CDR‐SB, ADL‐MCI, memory; CSF/MRI biomarkers	No benefit in primary endpoint; no clinical/CSF benefit; interpretation complicated by initial delivery‐device failure	Similar to placebo	Phase 2/3 completed	[135, 136, 137]
Insulin sensitizer	Metformin	Nondiabetic MCI and mild AD	Metformin or placebo for 8 weeks in crossover design	Placebo crossover	ADAS‐Cog, imaging, and plasma/CSF biomarkers	Very small pilot; not evidence of clinical efficacy. Proteomic analysis found changes in multiple plasma/CSF proteins.	Gastrointestinal symptoms, weight loss, elevated plasma lactate	Pilot Phase 2	[138]

*Abbreviations*: Aβ, amyloid‐β; AD, Alzheimer's disease; MCI, mild cognitive impairment; CDR‐SB, Clinical Dementia Rating‐Sum of Boxes; ADAS‐Cog, Alzheimer's Disease Assessment Scale‐Cognitive Subscale; ADCOMS, Alzheimer's Disease Composite Score; ADCS‐ADL‐MCI, Alzheimer's Disease Cooperative Study Activities of Daily Living for Mild Cognitive Impairment; ARIA, amyloid‐related imaging abnormalities; ARIA‐E, ARIA with edema or effusions; ARIA‐H, ARIA with hemosiderin deposition; MMSE, Mini‐Mental State Examination; iADRS, Integrated Alzheimer's Disease Rating Scale; IM, intramuscular; IV, intravenous; NfL, rate of neurofilament light; GFAP, glial fibrillary acidic protein; CSF, cerebrospinal fluid; GLP‐1RA, glucagon‐Like peptide‐1 receptor agonist; CBF, cerebral blood flow.

Meanwhile, nanodrug delivery systems have emerged as a critical research direction in the translational study of AD therapy in recent years [16, 17]. The BBB restricts the entry of most exogenous drugs, antibodies, nucleic acid therapeutics, and natural bioactive components into the central nervous system (CNS). Conventional administration routes are frequently confronted with insufficient cerebral drug exposure, peripheral adverse reactions, and limited targeting efficiency. Nanoscale delivery systems can enhance drug stability and bioavailability. Through surface ligand modification, receptor‐mediated transcytosis (RMT), nose‐to‐brain pathway delivery, stimuli‐responsive release, and cell membrane biomimetic strategies, they effectively enhance the ability of drugs to cross the BBB and target lesioned brain regions, serving as a vital bridge linking basic mechanistic research and clinical translation in current AD therapeutic studies [18].

Against the above background, this review systematically summarizes the latest research evidence regarding AD pathogenesis, therapeutic advances, and nanodelivery applications. It focuses on analyzing pathological mechanisms including protein homeostasis imbalance, neuroimmune microenvironment abnormality, mitochondrial dysfunction, lipid metabolic disorders, insulin resistance, metal ion homeostasis disturbance, microbial mediation, BBB impairment, and glymphatic system dysfunction. Furthermore, it outlines the clinical progress of AD pharmacotherapy, covering traditional symptomatic treatment, anti‐Aβ disease‐modifying therapy (DMT), tau‐targeted therapy, active immunization vaccines, immune regulation, and metabolic intervention. The value of nonpharmacological interventions in symptom relief, functional maintenance, and long‐term comprehensive management is also discussed. Finally, this paper further reviews the research progress of nanodrug delivery systems in AD treatment, with an emphasis on their potential and limitations in BBB penetration, improving central targeted delivery, delivering anti‐Aβ/tau agents, regulating neuroinflammation and oxidative stress, and delivery of nucleic acid therapeutics. By integrating evidence of pathological mechanisms and therapeutic strategies, this review is expected to provide theoretical references for the early identification, precise classification, multitarget combined intervention, and whole‐course management of AD in the future.

## 2 Pathological Mechanisms of AD

With the advancement of research, the traditional amyloid cascade hypothesis can no longer fully elucidate the complex pathogenesis of AD [4, 5]. In particular, the discovery of genetic risk factors such as TREM2, APOE, CD33, and ABCA7, as well as the identification of cell subpopulations including disease‐associated microglia (DAM), disease‐associated astrocytes (DAAs), and lipid droplet‐accumulating microglia (LDAM), have expanded the research paradigm of AD from the conventional neuron‐centric theory to a coupled network pathological model covering protein pathology, immune inflammation, oxidative stress, metabolic disturbance, metal ion homeostasis, microbial dysbiosis, and circulatory disorders (Figure 1) [4, 5, 19, 20, 21, 22, 23].

**FIGURE 1:**
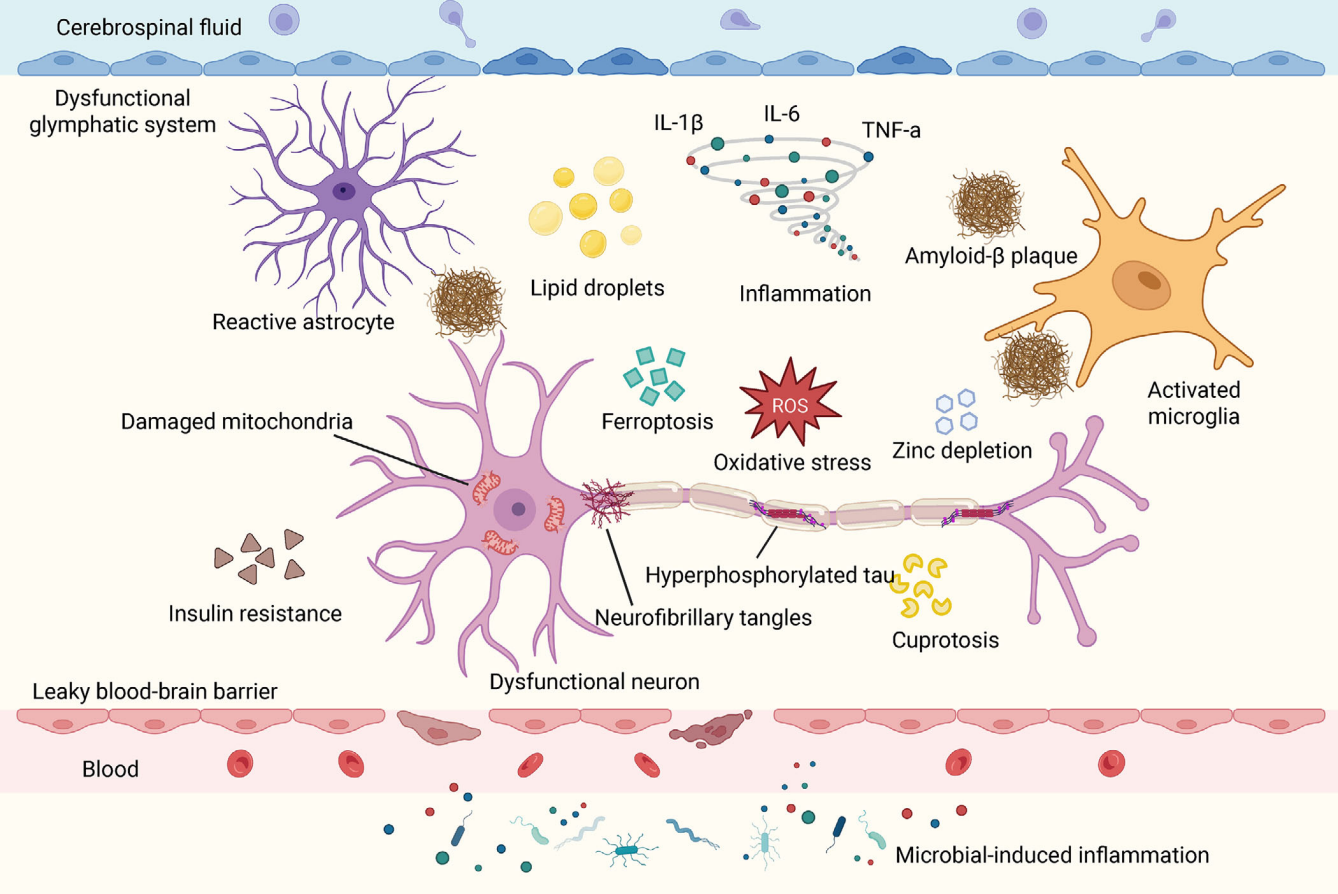
Pathogenesis of Alzheimer's disease. In recent years, the pathogenesis of AD has evolved from the traditional amyloid cascade hypothesis into an interconnected network pathological model involving protein pathology, immune inflammation, oxidative stress, metabolic disorder, metal ion homeostasis, microbial imbalance, and circulatory disturbance.

### 2.1 Proteostasis Homeostasis

The pathophysiological basis of AD is undergoing a paradigm shift from the traditional view of protein misfolding and deposition to the more sophisticated imbalance of proteostasis. Although the hypothesis centered on amyloid plaques and NFTs has dominated relevant research for decades [24, 25], accumulating evidence indicates that AD does not result from abnormalities in a single protein but rather represents a systemic imbalance collectively driven by the structural evolution, dynamic transformation, and synergistic interactions of multiple pathogenic proteins. This proteostasis imbalance is summarized into three key aspects: first, the dynamic polymorphism of Aβ challenges the previous understanding of its single toxic form, revealing a complex evolutionary process from unstable oligomers to pore‐forming annular assemblies [26, 27]. Second, the pathological driving effect of tau protein is no longer regarded as a secondary event downstream of Aβ. Through liquid–liquid phase separation (LLPS) and multisite posttranslational modifications (PTMs), tau exhibits distinct structural strain characteristics and prion‐like propagation capacity [28, 29, 30]. The recognition of TAR DNA‐binding protein 43 (TDP‐43) as an important additional proteinopathy beyond Aβ and tau provides a new perspective on the clinicopathological heterogeneity of AD. The crosstalk among TDP‐43, Aβ, and tau substantially accelerates the progression of neurodegeneration (Figure 2) [31, 32].

**FIGURE 2:**
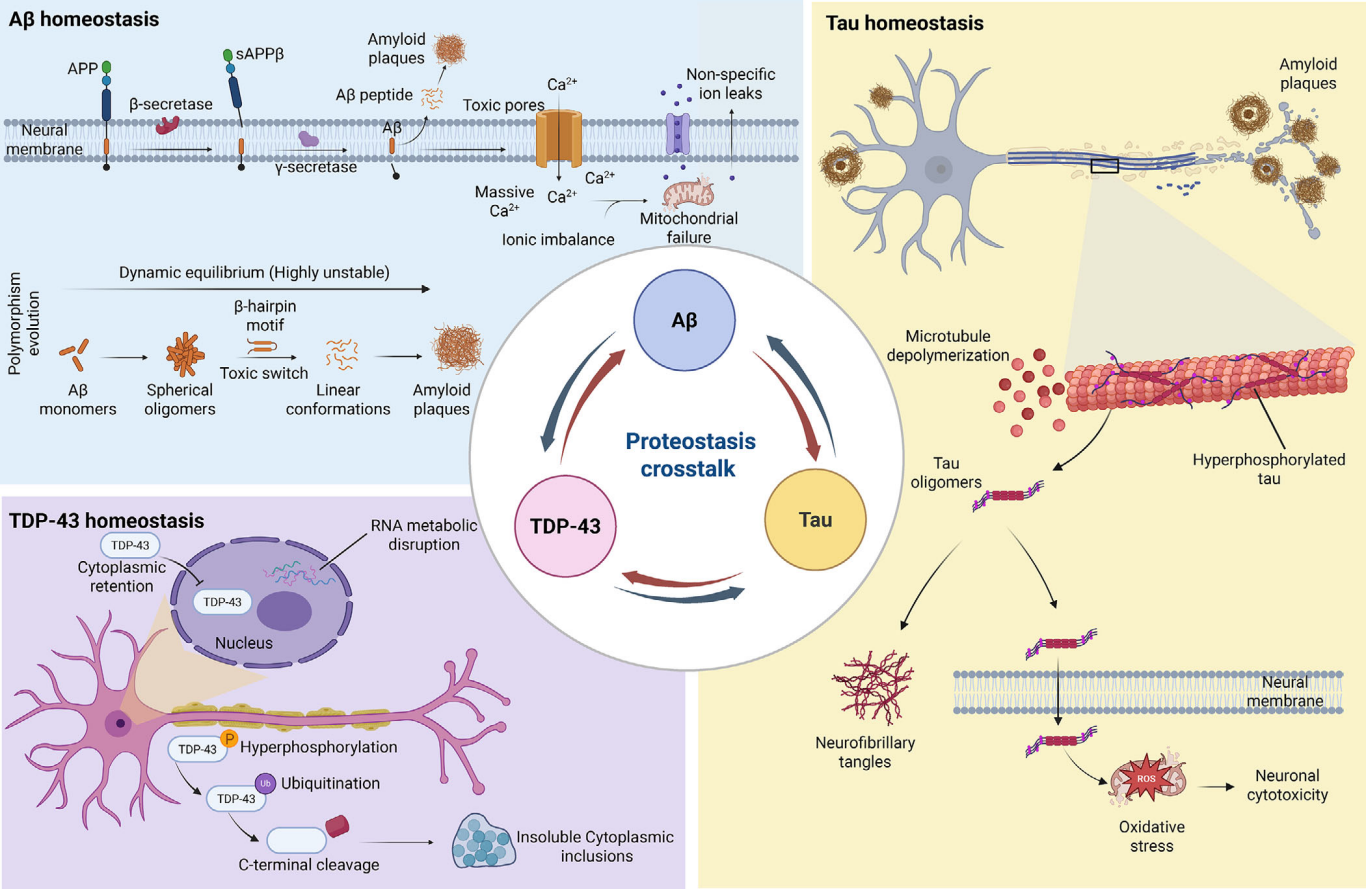
Proteostasis imbalance in Alzheimer's disease. The protein pathology of AD has evolved from simple misfolding and deposition of proteins into a more complex proteostasis imbalance, involving multiple pathogenic proteins such as Aβ, Tau, and TAR DNA binding protein 43 kDa (TDP‐43). The early formation of Aβ oligomers is a highly unstable dynamic process, in which the β‐hairpin motif acts as a crucial toxic trigger. Ring‐shaped annular assemblies of Aβ can form toxic pores, thereby inducing ionic homeostasis disruption and mitochondrial dysfunction. Tau undergoes liquid–liquid phase separation and multisite posttranslational modification to form polymorphic structural strains, which determine its differential toxicity and spreading propensity. Toxic Tau oligomers penetrate the cell membrane and trigger excessive reactive oxygen species (ROS) burst. Nuclear depletion of TDP‐43 leads to widespread disturbance of RNA metabolism and dynamic dysregulation of stress granules. *Abbreviation*: APP, amyloid precursor protein.

#### 2.1.1 Aβ Homeostasis

For decades, the Aβ cascade hypothesis has remained central to the pathological research of AD. This hypothesis proposes that aberrant Aβ deposition acts as the initiating event of AD pathogenesis, subsequently triggering NFTs, neuronal apoptosis, cerebrovascular damage and cognitive deterioration [2]. Aβ peptides are produced by abnormal cleavage of the transmembrane amyloid precursor protein (APP), which is critically involved in brain development, memory regulation, and synaptic plasticity maintenance [33, 34]. APP is sequentially cleaved by β‐secretase and γ‐secretase to generate Aβ fragments of varying lengths, which further assemble into neurotoxic oligomers and amyloid plaques [2]. The imbalance between Aβ production and clearance has long been recognized as a pivotal initiating factor of AD. In recent years, research focus has shifted toward the microstructural characteristics and toxic mechanisms of Aβ. With the application of high‐resolution approaches such as native mass spectrometry (native MS) and cryo‐electron microscopy (cryo‐EM), it has been well established that pathogenic Aβ oligomers are not structurally homogeneous aggregates, but dynamically evolving populations with marked structural polymorphism. At the early stage of oligomerization (from dimers to dodecamers), Aβ conformations maintain a highly unstable dynamic equilibrium. Cyclic ion mobility‐mass spectrometry monitoring reveals that Aβ undergoes prominent conformational rearrangement along with the increase of molecular weight at this stage [26]. The β‐hairpin motif within the Aβ sequence serves as a crucial toxic switch. Once beyond the tetramer stage, Aβ oligomers rapidly transform from innocuous spherical assemblies into highly neurotoxic linear conformations in a β‐hairpin‐dependent manner [35]. Increased polymerization complexity drives Aβ to form diversified structural branches. Cryo‐EM has directly captured dynamic Aβ42 oligomers with a molecular weight up to 150 kDa, which fail to mature into fibrils but assemble into higher‐order string‐like architectures [36]. These findings demonstrate that Aβ aggregation proceeds via multiple parallel pathways, and intermediate assemblies derived from distinct pathways exhibit differential neurotoxic potency and correlate with heterogeneous clinical phenotypes of AD.

After adopting specific toxic microconformations, Aβ induces substantial neuronal damage in a strictly structure‐dependent manner. Combined application of cryo‐electron tomography (cryo‐ET) and cryo‐EM has recently identified ring‐shaped annular Aβ assemblies. Insertion of these annular structures into neuronal lipid membranes forms nonspecific ion leakage pores, which trigger massive Ca^2^^+^ influx, thereby disrupting ionic homeostasis and inducing mitochondrial dysfunction [27]. In addition to structural polymorphism, PTMs of Aβ residues also profoundly modulate its neurotoxicity. For instance, nitration modification at the 10th tyrosine of Aβ1–42 inhibits Aβ aggregation and mitigates neurotoxicity [37]. Deamidation at asparagine 27 markedly attenuates Aβ aggregation propensity and alleviates microglia‐mediated neuroinflammatory responses [38].

#### 2.1.2 Tau Protein Homeostasis

Tau pathology is not merely a passive downstream event of Aβ, but possesses an independent driving mechanism. Under physiological conditions, tau acts as a microtubule‐associated protein (MAP) predominantly distributed in neuronal axons, which functions to stabilize microtubules and regulate axonal transport [39]. In the AD brain, the homeostatic balance of kinases is disrupted, leading to tau hyperphosphorylation. Hyperphosphorylated tau detaches from microtubules, thereby triggering microtubule depolymerization and axonal transport dysfunction. Dissociated phosphorylated tau monomers undergo misfolding and initially form soluble toxic oligomers. These species further aggregate into paired helical filaments, which eventually deposit as NFTs [6]. Similar to Aβ, the neurotoxicity and disease specificity of tau are collectively determined by its microstructural polymorphism, highly dynamic oligomeric evolution, and complex PTMs of amino acid residues. Direct visual evidence from cryo‐EM has confirmed that tau exhibits profound structural polymorphism and forms distinct pathological strains. Specific folding conformations expose unique molecular interfaces, which not only determine the seeding rate of endogenous normal tau, but also dictate aberrant interactions with specific organelles, ultimately resulting in regionally selective neuronal loss in a disease‐specific manner [28]. The transformation of tau from monomers to oligomers is a highly dynamic process. Pathological tau undergoes LLPS; such condensates maintain high internal dynamics and spontaneously assemble into toxic oligomers [40]. Tau oligomers readily penetrate cell membranes, induce mitochondrial dysfunction, disrupt calcium homeostasis, and trigger reactive oxygen species (ROS) burst, thereby exerting prominent neurocytotoxicity [41].

Notably, a recent study from the University of California has uncovered novel regulatory mechanisms underlying tau homeostasis via CRISPR screening in human induced pluripotent stem cell‐derived neurons. Specific signaling pathways, such as UFMylation and glycosylphosphatidylinositol anchoring, modulate tau oligomer formation. The E3 ubiquitin ligase complex serves as a critical regulator of tau abundance and is closely associated with resilience in tauopathies. This study further revealed that impaired mitochondrial function promotes aberrant proteolytic processing of tau, generating pathogenic fragments with high aggregation propensity [42]. Beyond canonical hyperphosphorylation, tau undergoes multiple motif modifications, including acetylation, ubiquitination, and isomerization. Mass spectrometry analyses have validated that acetylation at specific lysine residues (e.g., Lys174) not only weakens tau–microtubule binding affinity but also blocks ubiquitination at the same site. This modification directly impairs the proteasomal degradation of toxic tau species, leading to massive intracellular accumulation of pathological tau in neurons [29]. Isomerization represents a sophisticated nonenzymatic spontaneous modification. A recent prospective study investigated the effects of site‐specific isomerization within peptides of the tau R2 and R3 repeat domains. Age‐related d‐aspartate isomerization has been identified in aggregated tau from the AD brain. Such stereochemical alterations at specific residues markedly attenuate the capacity of tau seeds to induce full‐length tau fibrillation, indicating that spontaneous chemical modifications may serve as an intrinsic mechanism governing the propagation rate of tau pathology [30].

#### 2.1.3 TDP‐43 Homeostasis

TDP‐43 has emerged as another critical pathogenic protein, recognized as the third key molecule closely associated with AD following Aβ and tau. Traditionally, TDP‐43 is regarded as a pathological hallmark of amyotrophic lateral sclerosis and frontotemporal lobar degeneration. Nevertheless, accumulating postmortem and clinical evidence has demonstrated pathological TDP‐43 deposition in the brains of 30–70% of AD patients, which substantially accelerates cognitive decline [43, 44]. Under physiological conditions, TDP‐43 predominantly localizes in the neuronal nucleus. As an essential RNA‐binding protein, it participates in mRNA transcription, translation, splicing, and stability maintenance, as well as the biogenesis of microRNAs (miRNAs). During AD pathogenesis, impaired nucleocytoplasmic trafficking causes TDP‐43 to translocate from the nucleus and accumulate in the cytoplasm. The resultant nuclear loss‐of‐function triggers widespread disturbance of RNA metabolism, dynamic dysregulation of stress granules, and aberrant expression of pivotal genes, thereby impairing neuronal self‐repair and metabolic capacity [45]. Cytoplasmically retained TDP‐43 is highly susceptible to pathological PTMs, among which hyperphosphorylation (especially at Ser409/410), ubiquitination, and pathological C‐terminal truncation are the most prominent. These modifications drive misfolding of TDP‐43 monomers and their assembly into insoluble cytoplasmic inclusions. Such aggregates exert intrinsic neurotoxicity and severely disrupt mitochondrial function as well as the intracellular protein homeostasis network [46]. As the third pivotal pathological protein, TDP‐43 exhibits intensive crosstalk with Aβ and tau, the two core pathological markers of AD. Recent mechanistic studies have validated that abnormal TDP‐43 aggregation directly facilitates Aβ oligomerization and tau hyperphosphorylation. Conversely, the pathological microenvironment induced by Aβ and tau may also accelerate the mislocalization of TDP‐43 [31, 32]. Meanwhile, the low‐complexity sequence domains of tau and TDP‐43 interact via LLPS, enabling the two proteins to act reciprocally as scaffolds or clients and form cocondensates. Such multicomponent cocondensation rearranges molecular spatial organization and promotes irreversible deposition of pathological proteins [47]. The coexistence of multiple pathogenic proteins generates superimposed neurotoxicity. Consequently, AD patients with concomitant TDP‐43 pathology present more severe hippocampal sclerosis, hippocampal atrophy, and aggravated memory impairment compared with those without TDP‐43 lesions [48, 49]. Collectively, the essence of AD protein pathology lies not in the isolated deposition of a single abnormal protein, but in the cascade amplification resulting from simultaneous disruption of multiple protein homeostasis systems. Aβ, tau, and TDP‐43 represent three distinct pathological dimensions: extracellular amyloid aggregation, intracellular MAP dysfunction, and mislocalization of RNA‐binding proteins, respectively. These proteins mutually reinforce pathological progression through inflammatory responses, mitochondrial damage, LLPS, PTM, and impaired protein degradation pathways [26, 27, 28, 29, 30, 31, 32]. Therefore, future therapeutic strategies targeting the concurrent regulation of protein folding, phase separation, aberrant modification, intercellular propagation, and protein clearance systems may better accommodate the inherent complexity of AD protein pathology.

### 2.2 Neuroimmune Microenvironment

Neuroinflammation is no longer merely regarded as a secondary byproduct passively induced by Aβ deposition or tau NFTs; instead, it acts as one of the core initiating mechanisms driving the onset and progression of AD. This view has been strongly supported by recent genome‐wide association studies (GWAS) and single‐cell RNA sequencing (scRNA‐seq). Genetic evidence indicates that numerous high‐risk genes for late‐onset AD, such as TREM2, APOE, CD33, and ABCA7, are highly enriched in central immune cells [19, 23]. Furthermore, single‐cell and spatial transcriptomic technologies have precisely identified multiple disease‐specific cell subpopulations that determine disease progression [20]. Within this complex immune microenvironment, resident central glial cells (including microglia and astrocytes), together with their intricate molecular signaling pathways, jointly shape the evolutionary trajectory of AD (Figure 3).

**FIGURE 3:**
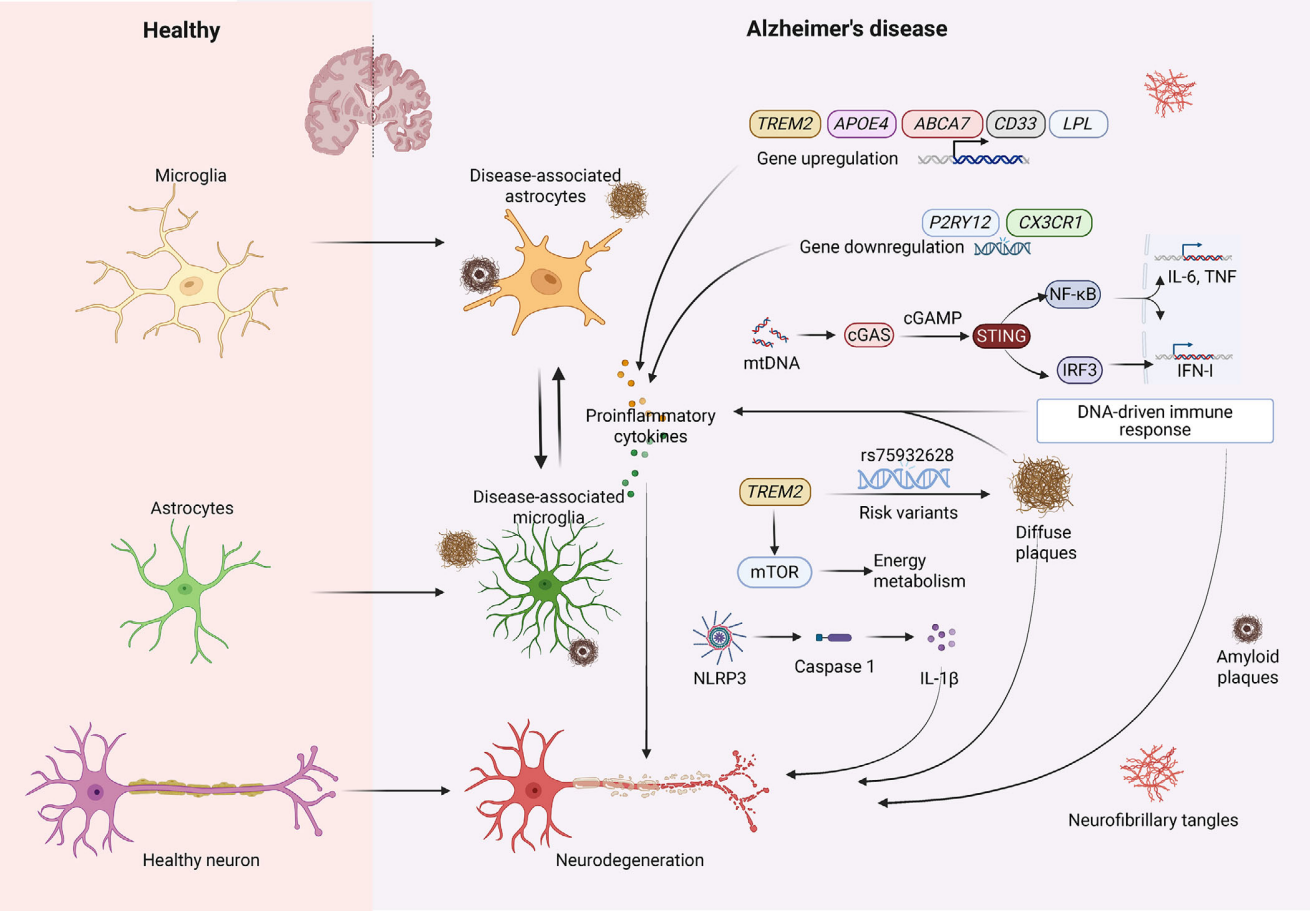
Neuroimmune microenvironment in Alzheimer's disease. Mutations in risk genes such as *TREM2*, *APOE*, *CD33*, and *ABCA7* occur in the AD brain. Sustained stimulation of the immune microenvironment is induced by amyloid plaques and neurofibrillary tangles, which drives microglia and astrocytes to transform into disease‐associated phenotypes and further triggers the activation of neuroinflammation. *Abbreviations*: NLRP3, NOD‐like receptor protein; mtDNA, mitochondrial DNA.

#### 2.2.1 Proinflammatory Phenotypic Transition of Microglia

Microglia are the resident innate immune cells of the CNS. In the early stage of AD, microglia exert neuroprotective effects by recognizing and clearing Aβ via phagocytic receptors [50]. However, sustained accumulation of pathological proteins triggers transcriptional reprogramming in microglia, driving their transition into DAM. DAM are characterized by the downregulation of homeostatic genes (e.g., P2ry12, Cx3cr1) and the upregulation of lipid metabolism‐related genes (e.g., Trem2, Apoe, LpL) [21]. This phenotypic shift is frequently accompanied by the acquisition of a proinflammatory profile, with excessive release of ROS and proinflammatory cytokines, which not only injure neurons but also accelerate the pathological propagation of AD‐related toxic proteins [51]. Cutting‐edge technologies such as scRNA‐seq have profoundly delineated the molecular trajectory underlying microglial conversion toward DAM. For instance, dysregulated expression of lysosomal β‐hexosaminidase A leads to ganglioside GM3 accumulation in microglial lysosomes, directly impairing Aβ phagocytosis and exacerbating neuroinflammation [52]. Spatially, microglial pathological responses exhibit marked regional heterogeneity. Region‐specific studies have revealed distinct patterns of microglia–neuron crosstalk: the THY1–ITGAX/ITGB2 signaling axis is activated in the prefrontal and occipital cortex, whereas the protein tyrosine phosphatase receptor type M(PTPRM) pathway dominates in the hippocampus. These findings highlight the necessity of brain‐region‐specific strategies for immune intervention [53]. Epigenetic mechanisms also participate in and amplify proinflammatory microglial phenotypes. Integrated transcriptomic analysis and experimental validation have demonstrated that histone H3K18 crotonylation (H3K18cr) promotes signal transducer and activator of transcription 1(STAT1) expression, thereby driving proinflammatory microglial polarization and ultimately contributing to cognitive impairment [54]. Meanwhile, Aβ stimulation rewires microglial metabolism from efficient oxidative phosphorylation toward less efficient but rapidly responsive aerobic glycolysis. Metabolic reprogramming is governed by the mTOR–HIF‐1α axis, which supplies adequate ATP and biosynthetic precursors for rapid proinflammatory cytokine production and cellular migration. Nevertheless, persistent hyper‐glycolysis cannot be sustained throughout disease progression, eventually resulting in innate immune tolerance and metabolic dysfunction in microglia [55].

#### 2.2.2 Functional Remodeling of Astrocytes

Similar to microglia, astrocytes in AD undergo profound transcriptional reprogramming and functional remodeling, shifting from a homeostatic physiological phenotype to DAAs [56]. Such remodeling leads to the loss of homeostatic supportive functions and the acquisition of neurotoxic properties in astrocytes. With disease progression, key molecules in astrocytes exhibit aberrant spatial distribution. For example, the loss of polarized aquaporin‐4 (AQP4) localization on astrocytic vascular endfeet severely impairs fluid circulation within the brain glymphatic system. This reduces the clearance efficiency of soluble Aβ and consequently accelerates extracellular Aβ deposition [57]. On the other hand, under persistent stimulation of Aβ plaques and chronic neuroinflammation, excessively activated microglia secrete inflammatory mediators including IL‐1α, TNF, and C1q, thereby inducing astrocytes to transform into neurotoxic A1‐type reactive phenotypes [58]. Remodeled astrocytes not only lose physiological supportive capacities such as promoting synaptogenesis, axonal outgrowth, and neurotransmitter uptake, but also actively release neurotoxic factors that trigger the death of neurons and mature oligodendrocytes. Notably, astrocytes also act as metabolic sensors that respond to systemic metabolic stress and modulate cerebral energy homeostasis; their dysfunction directly exacerbates energy metabolic crisis in the AD brain [59]. At the molecular regulatory level, aberrant activation of intracellular signaling cascades such as the JAK2/STAT3 axis serves as a core hub driving and sustaining the pathological reactive state of astrocytes. Inhibition of excessive pathway activation effectively reverses pathological astrocytic remodeling, markedly alleviates Aβ deposition, and rescues synaptic and cognitive deficits [60]. Accordingly, functional remodeling of astrocytes represents one of the central mechanisms directly driving synaptic loss and neurodegeneration.

#### 2.2.3 Core Molecular Pathways and Mechanisms Driving Neuroinflammation

Neuroinflammation is actively driven by multiple canonical signaling axes, including the NOD‐like receptor pyrin domain‐containing 3(NLRP3) inflammasome, triggering receptor expressed on myeloid cells 2(TREM2), cyclic GMP–AMP synthase (cGAS)–stimulator of interferon genes (STING), and the complement system.

As a central sensor of innate immunity, persistent activation of the NLRP3 inflammasome is well recognized as a pivotal driver of AD pathogenesis. Upon sensing aggregated Aβ or damage‐associated molecular patterns (DAMPs) released from injured cells via Toll‐like receptors (TLRs), microglia initiate NLRP3 inflammasome assembly and activation. This cascade facilitates Caspase‐1‐mediated maturation and secretion of proinflammatory cytokine IL‐1β, triggering chronic neuroinflammation [61]. Importantly, NLRP3 activation forms a malignant positive feedback loop in AD pathology. On one hand, extracellularly secreted apoptosis‐associated speck‐like protein containing a CARD (ASC) specks interact and cross‐seed with Aβ, acting as aggregation seeds to accelerate amyloid plaque deposition and spreading [62]. On the other hand, NLRP3‐related inflammatory signaling activates CaMKII‐α and GSK‐3β kinases, promoting tau hyperphosphorylation and NFT formation [63].

As a microglia‐specific receptor, TREM2 also plays an indispensable role in immune homeostasis regulation. GWAS have confirmed that the rare TREM2 variant rs75932628 (encoding R47H) is strongly associated with AD susceptibility [64]. Under physiological conditions, TREM2 signaling is essential for microglial migration toward plaques, formation of protective barrier envelopes, and efficient Aβ phagocytosis [21]. Loss‐of‐function or specific mutations of TREM2 disable microglia from assembling compact protective barriers around plaques, permitting plaque pathology dissemination and accelerating neurodegeneration [65]. Moreover, TREM2 maintains metabolic adaptability in microglia. By sensing ligands such as Aβ–lipoprotein complexes and membrane debris, TREM2 activates downstream mammalian target of rapamycin(mTOR) signaling to sustain high‐energy metabolic status. TREM2 deficiency drives microglia into metabolic stagnation and autophagic impairment, ultimately ablating their capacity to clear pathological substrates [66].

In recent years, the cytoplasmic double‐stranded DNA sensing cGAS–STING pathway has attracted increasing research attention. In the AD pathological microenvironment, damaged mitochondria in neurons and glial cells release mitochondrial DNA (mtDNA) into the cytoplasm [67, 68]. cGAS recognizes and binds ectopic cytoplasmic dsDNA, catalyzing the production of the second messenger cGAMP, which further activates STING. STING recruits and activates TANK‐binding kinase 1(TBK1) and interferon regulatory factor 3(IRF3) transcription factors, inducing robust expression of Type I interferons (IFN‐I) and IFN‐stimulated genes (ISGs), while triggering NF‐κB‐dependent inflammatory storms [69]. This mechanism directly couples cellular metabolic stress with neuroinflammation, constituting another critical driving force for AD progression.

Finally, aberrant activation of the complement system mediates pathological synaptic loss. As an initiator of the complement cascade, C1q is recruited by Aβ oligomers and deposited at postsynaptic sites. Subsequently, C1q activates downstream C3, which undergoes cleavage and accumulates on synaptic surfaces. Complement‐tagged synapses are recognized and excessively engulfed by complement receptor‐expressing microglia, leading to severe synaptic loss and cognitive decline prior to overt neuronal death [70]. Collectively, advances in neuroimmunology have reshaped the pathological framework of AD. Microglia and astrocytes are no longer regarded as passive responders secondary to Aβ and tau deposition; instead, they actively orchestrate disease progression through phagocytosis, antigen presentation, complement activation, cytokine release, and metabolic reprogramming [21, 50, 51, 58, 59, 60]. Notably, glial responses exhibit distinct temporal stages and dual‐phase characteristics. Therefore, future immunotherapeutic strategies should focus on restoring immune homeostasis rather than simply suppressing inflammation. This also explains the limited clinical efficacy of traditional broad‐spectrum anti‐inflammatory agents and suggests that precise modulation of TREM2, NLRP3, cGAS–STING, the complement system, and glial metabolic status holds greater translational potential for AD intervention.

### 2.3 Mitochondrial Dysfunction

Mitochondrial dysfunction is recognized as one of the early pathological hallmarks of AD, which occurs independently of Aβ deposition and even precedes amyloid plaque formation. According to the mitochondrial cascade hypothesis, individual mitochondrial genetic background and age‐related accumulated damage determine the susceptibility to AD [71]. Mitophagy serves as a critical quality‐control mechanism for cells to eliminate damaged mitochondria, and this process is severely disrupted in AD. Functional impairment of the PINK1/Parkin signaling pathway fails to enable timely recognition and autophagosomal recruitment of injured mitochondria, leading to aberrant accumulation of dysfunctional mitochondria in neurons. These impaired mitochondria continuously release ROS and ultimately trigger neuronal apoptosis [72, 73]. Meanwhile, Aβ and hyperphosphorylated tau exert direct toxic effects on mitochondria. Aβ translocates to the inner mitochondrial membrane and binds to amyloid‐binding alcohol dehydrogenase (ABAD), thereby interfering with the activity of respiratory chain complexes and impairing ATP production [74]. Tau disrupts microtubule‐dependent trafficking, hindering mitochondrial transport toward synaptic terminals and resulting in local energy depletion at synapses [75]. Of particular interest are mitochondria‐associated endoplasmic reticulum membranes (MAMs). MAMs not only regulate lipid synthesis and calcium homeostasis but also constitute an essential platform for APP processing. Dysfunction of MAMs induces lipid metabolic disorders, calcium overload, and subsequent neuronal death [76, 77]. As a key chaperone protein located on MAMs, Sigma‐1 receptor dysfunction is closely linked to mitochondrial defects and endoplasmic reticulum stress in AD, making Sigma‐1 receptor modulation a promising therapeutic strategy [78].

### 2.4 Dysregulated Lipid Metabolism

The brain is the organ with the highest lipid content in the human body, and disrupted cerebral lipid homeostasis represents a pivotal mechanism underlying AD pathogenesis. As the strongest genetic risk factor for late‐onset AD, apolipoprotein E4 (ApoE4) profoundly modulates cholesterol trafficking and distribution. Compared with the protective ApoE3 isoform, conformational alterations in ApoE4 reduce its lipidation capacity, impairing the efficient transport of cholesterol and phospholipids from astrocytes to neurons. This defect compromises synaptic plasticity and neuronal repair, while inducing abnormal intracellular cholesterol accumulation [79]. As an upstream regulatory hub, cholesterol metabolism independently modulates the aggregation of Aβ and tau in neurons [80]. On the one hand, neuronal cholesterol dyshomeostasis exacerbates proteostatic stress, attenuating protease‐mediated clearance of phosphorylated tau and facilitating its pathological accumulation. On the other hand, impaired cholesterol homeostasis directly alters APP processing via the cholesterol‐binding domain of APP, promoting excessive Aβ production [80]. Notably, recent RNA‐sequencing studies have identified a novel microglial subtype termed LDAM [22]. During AD progression, microglia phagocytose lipid‐rich myelin debris and Aβ plaques. However, functional deficiencies in ApoE4 or TREM2 hinder intracellular lipid catabolism, leading to massive cytoplasmic deposition of triglycerides and cholesterol esters as lipid droplets. Such lipid overload induces microglial phagocytic exhaustion and excessive proinflammatory cytokine secretion, forming a vicious immune‐metabolic positive feedback loop that accelerates neurodegeneration [81, 82].

### 2.5 Insulin Resistance

AD is increasingly regarded as a metabolic disorder, with cerebral insulin resistance and impaired glucose metabolism as core pathological features, earning AD the designation of “Type 3 diabetes” [83]. Distinct from systemic hyperglycemia, cerebral insulin resistance is defined as reduced neuronal sensitivity to insulin signaling [84]. Postmortem studies have demonstrated blunted insulin/IGF‐1 responsiveness in AD‐affected brain regions such as the hippocampus, accompanied by marked suppression of the IR/insulin receptor substrate 1 (IRS‐1)/PI3K signaling axis, mainly attributable to the inactivation of IRS‐1. Aberrant phosphorylation of IRS‐1 at multiple critical serine sites (e.g., Ser616, Ser636, Ser312) is positively correlated with oligomeric Aβ burden and negatively correlated with cognitive performance, indicating that Aβ oligomers may trigger abnormal IRS‐1 phosphorylation and drive the initiation and progression of cerebral insulin resistance [85]. Furthermore, impaired insulin signaling facilitates tau hyperphosphorylation. Under physiological conditions, Akt phosphorylates and inhibits GSK‐3β. In the context of insulin resistance, reduced Akt activity relieves the inhibitory constraint on GSK‐3β, leading to excessive GSK‐3β activation, robust tau phosphorylation, and subsequent NFT formation [86].

### 2.6 Dyshomeostasis of Metal Ions

Imbalanced metal ion homeostasis represents another pivotal pathogenic mechanism of AD. Aberrant distribution and altered concentrations of brain trace metals (primarily copper, zinc, and iron) not only exacerbate pathological protein aggregation but also trigger novel forms of regulated cell death. Synaptic redistribution and protein interaction of Cu^2^^+^ and Zn^2^^+^ directly facilitate amyloid plaque formation and synaptic dysfunction. In the AD pathological microenvironment, free copper and zinc are highly enriched within the synaptic cleft and act as high‑affinity ligands that directly bind APP and Aβ peptides [87, 88]. Zinc ions rapidly induce conformational rearrangement of Aβ and promote its precipitation. Consequently, zinc, which physiologically modulates synaptic plasticity, is largely sequestered within senile plaques, leading to local synaptic zinc depletion and impaired memory consolidation [88]. Meanwhile, copper binding to Aβ generates complexes with potent redox activity. These assemblies catalyze Fenton‑like reactions, continuously producing hydrogen peroxide and hydroxyl radicals, thereby provoking severe local oxidative stress and neuronal programmed cell death [89]. Furthermore, the catalytic effects of Cu^2^^+^ and Zn^2^^+^ on tau pathology have attracted substantial attention. Free Cu^2^^+^ and Zn^2^^+^ possess specific binding sites within the microtubule‑binding domain of tau, inducing and accelerating its conversion into neurotoxic β‑sheet oligomers. This markedly promotes tau aggregation and reshapes fibril morphology [87, 90, 91]. These findings reveal an essential bioinorganic linkage between metal dyshomeostasis and proteinopathy in AD.

In addition, pathological iron accumulation and ferroptosis have become research hotspots in neurodegeneration. Clinical neuroimaging and histological examinations have consistently demonstrated marked iron overload in the hippocampus and cerebral cortex of AD patients [89, 92]. Excessive intracellular iron upregulates APP expression at the translational level via iron‑responsive elements. More critically, labile iron drives robust lipid peroxidation of polyunsaturated fatty acids in neuronal membranes. When iron‑dependent lipid peroxide accumulation exceeds the scavenging capacity of the cellular antioxidant system such as glutathione peroxidase 4, ferroptosis is ultimately initiated [92]. Reciprocally, Aβ and hyperphosphorylated tau further promote cerebral iron deposition, forming a self‑amplifying vicious cycle that accelerates AD progression. Collectively, mitochondrial dysfunction, lipid metabolic disturbance, insulin resistance, and metal ion dyshomeostasis jointly indicate that AD is not merely a protein deposition disorder, but a multisystem disease characterized by profound metabolic homeostasis collapse. Mitochondrial impairment causes insufficient ATP production, excessive ROS release, and disrupted calcium balance. ApoE4‑mediated lipid dysregulation, impaired cholesterol trafficking, and microglial lipid droplet accumulation further compromise Aβ clearance, synaptic repair, and immune homeostasis [22, 71, 79, 80]. Cerebral insulin resistance bridges Aβ oligomer toxicity, GSK‑3β hyperactivation, and tau hyperphosphorylation [83, 85, 86]. Dysregulated copper, zinc, and iron accelerate neurodegeneration by facilitating Aβ/tau aggregation, aggravating oxidative stress, and triggering ferroptosis [87, 90, 91, 92]. Accordingly, future AD therapeutic strategies should also target the restoration of cellular energy supply, lipid metabolism, redox equilibrium, and metabolic signaling networks.

### 2.7 Microbial‐Mediated Pathogenesis

In recent years, the driving role of microorganisms, especially gut and oral microbiota, in AD pathogenesis has gained widespread recognition. Dysregulation of the microbiota–gut–brain axis and microbiota–oral–gut–brain axis is regarded as a crucial systemic factor promoting AD pathological progression.

#### 2.7.1 Gut Microbiota

AD patients exhibit significantly reduced gut microbial diversity, characterized by increased pathogenic bacteria and depleted beneficial commensal microbes [93]. First, bacterial toxins can cross the intestinal barrier and blood–brain barrier to initiate neuroinflammation. Aging and gut dysbiosis increase intestinal permeability, resulting in leaky gut syndrome [94]. Lipopolysaccharide (LPS) derived from Gram‑negative bacteria leaks into the peripheral circulation, triggering systemic inflammation, and compromising BBB integrity [95, 96]. Circulating LPS infiltrating the CNS binds TLR4 on microglia and induces robust NF‑κB‑driven neuroinflammation [97]. Second, gut dysbiosis depletes protective microbial metabolites and alters metabolic profiles. Short‑chain fatty acids (SCFAs), produced by anaerobic fermentation of dietary fiber, exert neuroprotective effects by mitigating neuroinflammation, promoting neurogenesis, enhancing synaptic plasticity, and maintaining BBB integrity. In AD, drastic depletion of SCFA‑producing bacteria abolishes this vital neuroprotective signaling [98]. Third, emerging evidence reveals profound crosstalk between gut microbiota and the AD risk gene APOE. Specific microbial communities accelerate tau pathology and neuroinflammation in an ApoE isoform‑dependent manner. Germ‑free mice or antibiotic‑treated AD mice exhibit markedly reduced disease‑associated microglial and astrocytic phenotypes, accompanied by alleviated Aβ plaque burden and tau‑driven neurodegeneration. These findings confirm the indispensable role of gut microbiota in driving neurotoxicity and glial pathological remodeling in AD [99].

#### 2.7.2 Oral Microbiota

Oral microbiota has emerged as a critical contributor to AD pathogenesis, with chronic periodontitis showing the most robust pathological correlation. Oral microbial dysbiosis, particularly overgrowth of periodontal pathogens, facilitates AD progression through multiple pathways.

First, periodontal pathogens and their virulence factors can directly invade the brain. Porphyromonas gingivalis is a key etiological agent of chronic periodontitis. A landmark study in 2019 detected P. gingivalis and its toxic gingipain proteases in brain tissue and cerebrospinal fluid (CSF) from AD patients. Gingipains promote Aβ1–42 generation and amyloid plaque deposition, while directly cleaving tau to induce its hyperphosphorylation and NFT formation [100]. Second, oral dysbiosis exacerbates central neuroinflammation via systemic inflammation and BBB disruption. Persistent immune activation in periodontal pockets releases abundant pro‑inflammatory cytokines (IL‑1β, IL‑6, TNF‑α) and bacterial LPS into the bloodstream. Peripheral inflammatory mediators disrupt endothelial tight junctions, increase BBB permeability, and activate microglia and astrocytes within brain parenchyma, triggering sustained neuroinflammatory storms [101]. Fusobacterium nucleatum is another periodontal pathogen, with elevated serum antibodies detected in AD patients. Recent studies have demonstrated that F. nucleatum directly activates microglia via the MyD88–MAPK pathway, aggravating neuroinflammation and cognitive impairment in AD model mice [102]. Finally, emerging evidence highlights the indirect cascade regulation of the oral–gut–brain axis. Excessively proliferated oral pathogens enter and colonize the gastrointestinal tract via swallowing, disrupting intestinal microecological balance. For instance, oral infection with F. nucleatum reshapes gut microbial composition, further exacerbating cognitive deficits and AD pathological features in model rats through intestinal immune disturbance and metabolic dysfunction [103]. This dual‑axis regulatory mechanism suggests that targeting the oral microbiome represents a promising novel window for early AD intervention.

### 2.8 Blood–Brain Barrier Dysfunction

Structural disruption and functional impairment of the BBB constitute another critical pathological hallmark of AD. Under physiological conditions, endothelial cells, pericytes, astrocytes, and neurons collectively form the neurovascular unit (NVU), which maintains barrier integrity and regulates substance exchange between the circulation and the brain parenchyma. In AD, BBB dysfunction triggers NVU structural remodeling, disrupts the influx and efflux transport of Aβ and glucose, and facilitates the leakage of blood‐derived proteins into the brain [104]. First, imbalanced receptor‐mediated transmembrane transport in brain endothelial cells impairs Aβ clearance. Low‐density lipoprotein receptor‐related protein 1 (LRP1) mediates Aβ efflux from the brain parenchyma into the peripheral circulation, whereas the receptor for advanced glycation end products (RAGE) promotes peripheral Aβ influx into the brain. In AD, significant downregulation of LRP1 and upregulation of RAGE lead to impaired Aβ efflux and excessive peripheral influx, directly elevating cerebral Aβ burden [105]. Pathological Aβ deposition within cerebral vessel walls further induces cerebral amyloid angiopathy (CAA). The resultant prothrombotic tendency and microvascular injury exacerbate cerebral hypoperfusion and hypoxic damage, thereby accelerating neurodegeneration [106]. Degeneration and loss of brain capillary pericytes exacerbate neuronal stress induced by leakage of blood‐borne neurotoxic molecules. Neuroimaging studies have confirmed prominent BBB impairment in APOE4 carriers. Genetic knock‐in animal evidence demonstrates that the APOE4 allele activates the cyclophilin A–matrix metalloproteinase‐9 proinflammatory cascade in pericytes, triggering tight junction protein degradation and pericyte apoptosis, which further deteriorates neuronal and synaptic function [107]. Compromised BBB integrity allows massive leakage of circulating neurotoxic macromolecules such as fibrinogen, thrombin, albumin, and immune cells into the brain parenchyma. In particular, leaked fibrinogen converts into insoluble fibrin deposits, which potently activate microglia surrounding amyloid plaques. Such microglial hyperactivation drives excessive release of ROS and proinflammatory cytokines, establishing a self‐sustaining vicious cycle of oxidative stress and chronic neuroinflammation [108].

### 2.9 Glymphatic System Dysfunction

The cerebral glymphatic system represents an astrocyte‐mediated dynamic circulatory network that relies on CSF–brain interstitial fluid (ISF) exchange to clear metabolic waste and neurotoxic proteins, predominantly activated during sleep. Physiological rhythmic activities, including cardiovascular pulsation, respiratory oscillation, and neural oscillation, coordinately modulate glymphatic clearance efficiency [109]. Accumulating evidence indicates that glymphatic dysfunction is prevalent in AD patients, hindering the clearance of neurotoxic proteins such as Aβ and tau and promoting their intracerebral aggregation [110, 111]. Glymphatic impairment arises from multiple intertwined pathological mechanisms. First, mislocalization and loss of AQP4 distribution serve as the core molecular driver of glymphatic failure. Physiologically, AQP4 is highly enriched on astrocytic vascular endfeet, and this polarized localization is essential for driving CSF–ISF bulk flow. In the AD pathological microenvironment, reactive astrocytes undergo AQP4 depolarization, with abnormal redistribution from vascular endfeet toward the soma, which drastically reduces the clearance efficiency of interstitial Aβ and tau [112, 113].

Second, diminished arterial pulsatility and increased vascular resistance weaken hydrodynamic driving force for glymphatic perfusion. Glymphatic fluid influx into the brain parenchyma is highly dependent on mechanical pulsation of penetrating arteries. Aging and concomitant CAA in AD cause vascular stiffening and pericyte loss, markedly attenuating arterial pulsatility and impairing CSF perfusion into brain tissues [109, 114]. Third, circadian rhythm disturbance and pathological protein deposition form a reciprocal vicious cycle. Glymphatic clearance peaks during slow‐wave sleep, whereas AD patients frequently suffer from severe sleep disorders. Disrupted sleep deprives the brain of optimal waste clearance windows and disrupts the diurnal balance of Aβ production and clearance; uncleared Aβ further damages sleep‐regulating neural circuits [114]. Fourth, progressive degeneration of downstream meningeal lymphatics obstructs waste drainage outflow. Emerging studies have linked the intracerebral glymphatic system to extracranial lymphatic networks. Human magnetic resonance imaging has identified major lymphatic structures within the dura mater, with lymphatic pathways along cranial nerves and blood vessels directly connecting to cervical lymph nodes [115]. Waste‐laden ISF drains from the brain parenchyma into meningeal lymphatics and is ultimately eliminated via cervical lymph nodes and systemic circulation. In AD, progressive deterioration of dural lymphatic vasculature impairs the drainage of cerebral metabolic waste to cervical lymph nodes. Obstructed downstream outflow increases upstream glymphatic drainage resistance, trapping neurotoxic metabolites in the brain and perpetuating neuroinflammation and pathological protein propagation [109].

Overall, microbial dysbiosis, BBB disruption, and glymphatic dysfunction broaden the understanding of AD pathogenesis to a systemic perspective. Beyond pathological protein deposition, AD should be recognized as a systemic brain network disease involving peripheral inflammation, vascular dysfunction, and impaired cerebral clearance systems. Gut and oral microbiota modulate the central immune microenvironment through bacterial toxins, depleted protective metabolites, periodontal pathogens, and systemic inflammatory signaling [94, 95, 96, 97]. BBB breakdown facilitates the infiltration of peripheral inflammatory mediators, blood‐derived proteins, and immune cells into the brain parenchyma, further amplifying neuroinflammation [104, 105, 106, 107, 108]. Glymphatic insufficiency hinders the clearance of Aβ, tau, and metabolic waste, creating a vicious cycle linking protein deposition, sleep disturbance, reduced vascular pulsatility, and meningeal lymphatic degeneration [110, 111, 112, 113, 114, 115]. These insights suggest that future AD prevention and treatment should attach greater importance to intervention strategies beyond conventional neuropharmacology, including oral health maintenance, intestinal microecological regulation, vascular protection, sleep management, and preservation of cerebral clearance function.

## 3 Clinical Advances in Pharmacological Therapy for AD

As a complex neurodegenerative disorder, AD has witnessed a long and tortuous journey in drug research and development. For decades, AD pharmacological management has mainly relied on symptomatic agents including ChEIs and NMDA receptor antagonists [6]. Although these medications can alleviate clinical symptoms to a certain extent, they are incapable of reversing or halting disease pathological progression. In recent years, with an in‐depth understanding of AD biological mechanisms, the landscape of drug development has undergone a remarkable shift, evolving from mere symptomatic management toward DMTs that alter disease trajectory [116]. In particular, the approval of anti‐Aβ monoclonal antibodies has ushered in a new era of precision immunotherapy for AD [7, 8]. Nevertheless, single‐target therapies have exhibited inherent limitations in clinical practice, driving research attention toward broader pathological mechanisms, including tau pathology, active immunization, neuroinflammation, and immune regulation [11, 12, 13]. Meanwhile, drug repurposing of antidiabetic agents offers cost‐effective and safe alternative strategies for AD intervention (Figure 4) [14, 15].

**FIGURE 4:**
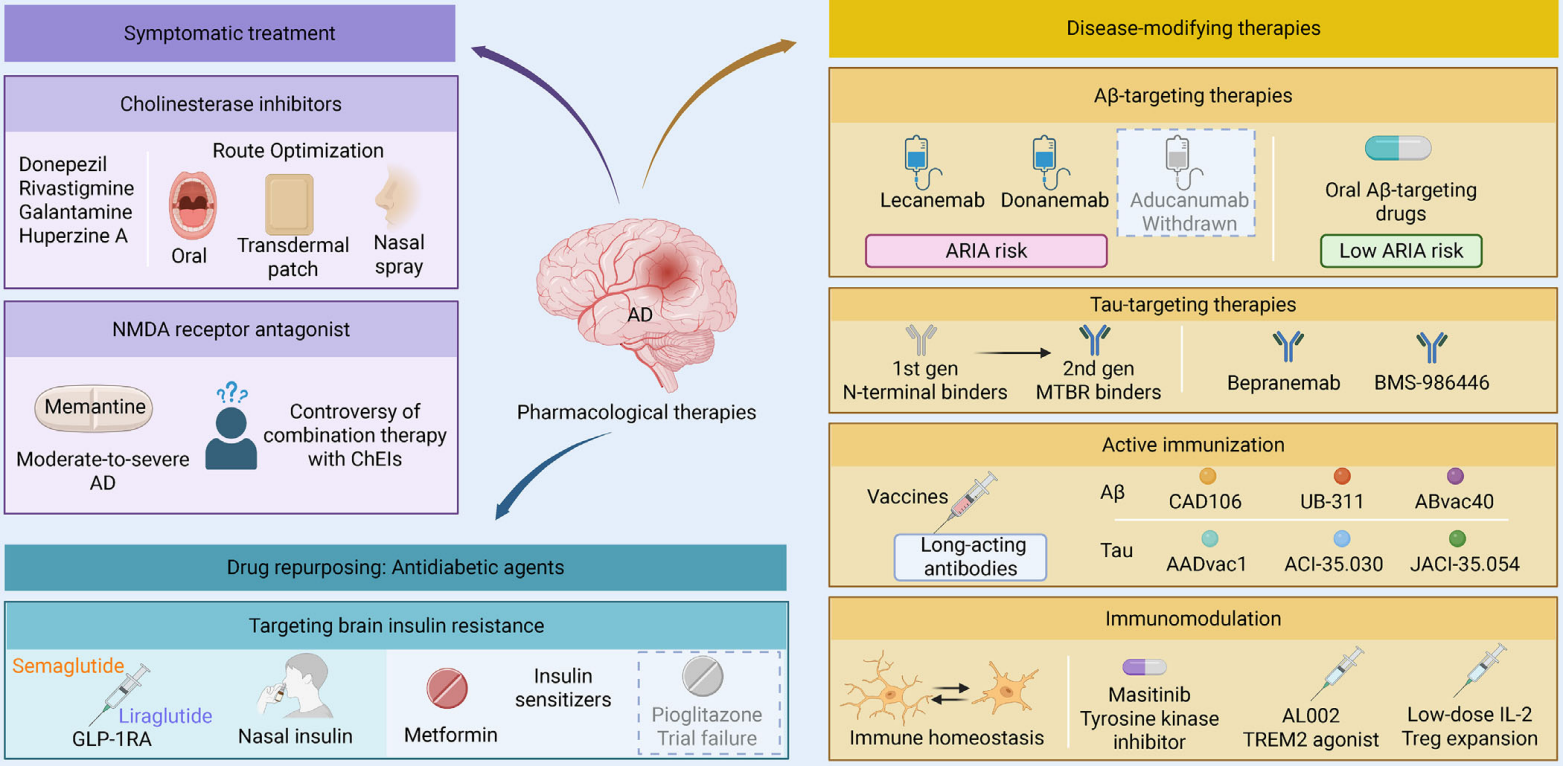
New advances in pharmacological therapy of Alzheimer's disease. The clinical pharmacological treatment of Alzheimer's disease (AD) has evolved from symptomatic therapy to disease‐modifying therapy, including Aβ monoclonal antibodies, Tau monoclonal antibodies, active immune vaccines, and immunomodulation. In addition, antidiabetic drugs exhibit unique therapeutic potential. *Abbreviations*: GLP‐1RA, glucagon‐like peptide 1 receptor agonists; ARIA, amyloid‐related imaging abnormalities.

### 3.1 Cholinesterase Inhibitors

ChEIs remain first‐line pharmacological options for mild‐to‐moderate AD, exerting predominant benefits in symptomatic relief and functional deterioration delay. Clinically available ChEIs include donepezil, rivastigmine, galantamine, and huperzine A. Contemporary research on ChEIs has focused on optimizing clinical application strategies. On the one hand, intensified regimens have been explored for moderate‐to‐severe AD. Clinical experience with 23 mg/d donepezil indicates sustained therapeutic value in advanced AD patients, with close attention required for dose titration, gastrointestinal adverse event monitoring, patient selection, and individualized dose adjustment [139]. On the other hand, long‐term clinical outcomes of ChEI administration have been validated in longitudinal cohort studies. ChEI use is associated with slower cognitive decline, reduced mortality risk, and lower incidence of severe dementia. Over a 5‐year follow‐up period, ChEI users maintained higher Mini‐Mental State Examination (MMSE) scores at each visit, with an annual gain of 0.13 points (95% CI: 0.06–0.20). Compared with nonusers, ChEI administration reduced mortality risk by 27% (HR = 0.73, 95% CI: 0.69–0.73). Among all agents, galantamine was linked to a lower risk of severe dementia (HR = 0.69, 95% CI: 0.47–1.00) [140].

The development of novel formulations represents another practical progress, aiming to improve medication adherence and tolerability. Transdermal patches and nasal sprays are the two major emerging dosage forms. Rivastigmine transdermal patch is the first ChEI formulated for percutaneous delivery. Recent systematic reviews have demonstrated that rivastigmine patches significantly reduce gastrointestinal adverse events compared with oral formulations, offering particular advantages for patients with dysphagia, poor adherence, or difficult caregiver management [141]. Subsequently, donepezil transdermal patches have become a research and translational hotspot. In 2022, Adlarity became the first donepezil transdermal patch approved by the United States Food and Drug Administration (US FDA) for AD treatment [142]. A multinational, multicenter, randomized, double‐blind, active‐controlled Phase 3 clinical trial evaluated the efficacy, and safety of donepezil transdermal patches. After 24 weeks of treatment, no statistically significant differences were observed between transdermal and oral donepezil in full‐itemized Alzheimer's Disease Cooperative Study Activities of Daily Living(ADCS‐ADL), Clinician Interview‐Based Impression, MMSE, Neuropsychiatric Inventory (NPI), and Clinical Dementia Rating (CDR). Although local pruritus and erythema occasionally occurred, the patch formulation remained well tolerated. Assessments of adverse events, vital signs, and laboratory parameters confirmed comparable safety profiles between transdermal and oral donepezil [143]. Additionally, a novel rivastigmine nasal spray exhibits superior bioavailability, rapid onset, and fewer side effects relative to transdermal delivery [144].

### 3.2 NMDA Receptor Antagonists

NMDA receptor‐mediated excitotoxicity plays a pivotal role in AD‐related neurodegeneration. Memantine, a low‐to‐moderate affinity noncompetitive NMDA receptor antagonist, is the most established glutamatergic modulator currently applied in routine AD clinical practice. Memantine is US FDA‐approved for moderate‐to‐severe AD but not yet indicated for early‐stage disease. Multiple randomized, double‐blind, placebo‐controlled trials have verified that memantine improves cognitive performance, ADL, global function, and neuropsychiatric symptoms in patients with moderate‐to‐severe AD [145, 146, 147]. A recent study adopted 24‐h sleep deprivation in healthy volunteers to simulate working memory deficits resembling AD‐related cognitive impairment. The results showed that 15‐day memantine supplementation significantly improved reaction time and working memory performance [148]. Moreover, a recent Phase 3 trial in patients receiving whole‐brain radiotherapy demonstrated that memantine preserved cognitive function and alleviated patient‐reported symptoms [149].

For patients progressing to moderate‐to‐severe AD under stable ChEI maintenance, optimal therapeutic adjustment remains controversial, including whether to continue single ChEI therapy, switch to memantine monotherapy, or adopt ChEI–memantine combination treatment. Previous studies have yielded inconsistent conclusions. A randomized controlled trial (RCT) by Tariot et al. reported that adding memantine to stable donepezil treatment conferred superior benefits in cognition, daily living ability, global outcome, and behavioral symptoms relative to placebo in moderate‐to‐severe AD patients [146]. Subsequent meta‐analyses further confirmed that combined ChEI and memantine therapy delays cognitive deterioration more effectively in moderate‐to‐severe AD [150, 151, 152]. In December 2014, the US FDA approved Namzaric, a fixed‐dose combination of memantine and donepezil, for moderate‐to‐severe AD [153]. The 2015 EFNS‐ENS/EAN guidelines also recommended ChEI plus memantine combination therapy for this patient population [154].

Nevertheless, doubts persist regarding the incremental benefits of combined over monotherapy due to insufficient high‐level clinical evidence [155]. The DOMINO trial published in 2012 indicated no additional clinical gain from combination treatment. Compared with donepezil placebo, continued donepezil monotherapy yielded a 1.9‐point higher Standardized MMSE (SMMSE) score (95% CI: 1.3–2.5; P<0.001) and a 3.0‐point lower Bristol Activities of Daily Living Scale (BADLS) score (indicating milder functional impairment; 95% CI: 1.8–4.3; *p* < 0.001). Switching to memantine monotherapy also produced significant improvements versus memantine placebo, with a 1.2‐point SMMSE elevation (95% CI: 0.6–1.8; *p* < 0.001) and a 1.5‐point BADLS reduction (95% CI: 0.3–2.8; *p* = 0.02). Notably, no statistically significant differences were identified in primary endpoints (SMMSE, BADLS) or secondary outcomes (NPI, DEMQOL‐Proxy, caregiver health status) between donepezil monotherapy and donepezil–memantine combination therapy [156]. Recent meta‐analyses further supported that combination treatment provides no additional clinical benefits over monotherapy in moderate‐to‐severe AD [157]. Accordingly, the clinical value of ChEI–memantine combined therapy remains highly debatable.

Overall, although ChEIs and memantine cannot modify the core pathological cascade of AD, their clinical roles should not be completely replaced by emerging DMTs. These conventional agents possess distinct advantages including abundant clinical experience, broad applicability, relatively low cost, and proven efficacy in ameliorating cognitive, behavioral and functional deficits [139, 140, 145, 146, 147]. However, their therapeutic effects are limited to symptomatic relief and functional maintenance, with little capacity to halt irreversible neurodegeneration. Therefore, amid the gradual clinical translation of DMTs, traditional symptomatic medications should serve as foundational components of comprehensive long‐term AD management rather than being mutually substituted with disease‐modifying strategies.

### 3.3 Aβ‐Targeted DMTs

DMTs refer to interventions that alter the fundamental pathophysiological processes of AD and exert beneficial effects on disease trajectory [116]. Given that cerebral Aβ plaque deposition constitutes the core pathological hallmark of AD, clearance or reduction of Aβ based on the amyloid cascade hypothesis has become a pivotal DMT strategy. In recent years, multiple intravenously administered anti‐Aβ monoclonal antibodies have been shown to markedly reduce brain amyloid burden and slow cognitive decline to a certain extent, marking a paradigm shift from pure symptomatic management to disease‐modifying treatment for AD. Anti‐Aβ monoclonal antibodies are categorized into first‐generation agents (bapineuzumab and solanezumab) and second‐generation counterparts (aducanumab, lecanemab, and donanemab). Bapineuzumab was one of the earliest anti‐Aβ antibodies to enter Phase 3 trials, targeting the N‐terminal epitope of Aβ. Phase 3 results demonstrated that although bapineuzumab reduced cerebral amyloid load, it failed to yield significant improvements in cognitive or functional outcomes, leading to eventual discontinuation of development. Nevertheless, these trials validated the feasibility of brain Aβ clearance and clarified the risk of amyloid‐related imaging abnormalities (ARIA) [158]. Solanezumab primarily binds soluble Aβ monomers. Two pivotal Phase 3 studies confirmed that solanezumab could not attenuate cognitive and functional deterioration, nor did it achieve statistically significant amyloid reduction [159, 160], resulting in regulatory nonapproval. Currently, second‐generation anti‐Aβ monoclonal antibodies dominate clinical practice, mainly indicated for mild cognitive impairment (MCI) due to AD and mild AD dementia. Aducanumab targets aggregated Aβ species and received accelerated US FDA approval in 2021, yet remained controversial owing to inconsistent Phase 3 outcomes. Using the CDR ‐ Sum of Boxes(CDR‐SB) as the primary endpoint, the EMERGE trial met its predefined endpoint (difference, −0.39; 95% CI, −0.69 to −0.09; *p* = 0.012), whereas the ENGAGE trial did not (difference, 0.03; 95% CI, −0.26 to 0.33; *p* = 0.83) [161]. Ultimately, aducanumab was voluntarily withdrawn from the market in January 2024 amid ongoing controversy.

Lecanemab selectively targets soluble Aβ protofibrils. The Phase 3 Clarity AD trial revealed that lecanemab slowed CDR‐SB progression by 27% (difference, −0.45; 95% CI, −0.67 to −0.23; *p* < 0.001) and substantially reduced brain amyloid burden (difference, −59.12 centiloids; 95% CI, −62.64 to −55.60; *p* < 0.001), accompanied by statistically significant improvements in cognition, function and daily living performance. ARIA with edema or effusion occurred in 12.6% of recipients, highlighting the necessity of regular MRI surveillance [117]. In 2023, lecanemab obtained full US FDA approval, becoming the first fully authorized anti‐Aβ monoclonal antibody for AD DMT. Recent real‐world reports further indicated potential risks of elevated d‐dimer levels, thromboembolic events and severe urinary retention, emphasizing the need for rigorous postmarketing safety monitoring [7, 118].

Donanemab, as an antibody targeting modified Aβ deposited, has shown potential to improve integrated AD Rating Scale in early AD patients (difference, 2.92; 95% CI, 1.51–4.33; *p* < 0.001) [9]. The subsequent Phase 3 trial confirmed robust amyloid clearance superior to aducanumab. At 6, 12, and 18 months, amyloid plaque clearance rates reached 37.9, 70.0, and 76.8% in the donanemab group, respectively. The median clearance time was 359 days for donanemab versus 568 days for aducanumab (*p* < 0.001) [10]. Notably, donanemab carries a substantial ARIA risk of 36.8%, representing a critical safety concern [9]. Even so, donanemab was approved by the US FDA in July 2024 as another second‐generation DMT following lecanemab. Recent systematic reviews concluded that both lecanemab and donanemab improve cognitive and functional outcomes in early AD, while rigorous safety surveillance and biomarker‐based patient screening (Aβ PET or CSF profiling) are indispensable [8].

To overcome limitations of large‐molecule antibodies, including low blood–brain barrier permeability and inconvenient intravenous administration, oral small‐molecule agents have emerged as a promising developmental direction. ALZ‐801 (valiltramiprosate), an oral small‐molecule prodrug targeting Aβ oligomers, exhibits distinct translational advantages. As a valine‐conjugated prodrug of tramiprosate, ALZ‐801 is rapidly metabolized into active tramiprosate and its endogenous metabolite 3‐sulfopropionic acid (3‐SPA) after oral intake. 3‐SPA is natively present in the human brain and effectively inhibits the assembly of toxic oligomers from Aβ42 monomers [162]. Clinical pharmacokinetics have verified excellent oral bioavailability and favorable brain penetration of ALZ‐801, with steady‐state exposure well maintained in APOE4 carriers and unaffected by age, gender, or concomitant medications [163, 164]. In a Phase 2 trial focusing on APOE4 carriers with early AD, ALZ‐801 significantly reduced plasma phosphorylated tau (p‐Tau) levels, attenuated hippocampal atrophy, and exhibited no incidence of ARIA with vasogenic edema (ARIA‐E), demonstrating a more favorable safety profile than monoclonal antibodies [119, 120]. Quantitative systems pharmacology further validated that ALZ‐801 counteracts the natural decline trajectory of CSF and plasma amyloid biomarkers and stabilizes cognitive function, with more prominent efficacy at the MCI stage [165]. These findings support the broad prospect of precision oral antiamyloid strategies tailored to specific genetic subgroups.

The clinical breakthrough of anti‐Aβ therapy represents a landmark milestone in AD management; nevertheless, current evidence calls for rational evaluation of Aβ‐targeted strategies. The success of lecanemab and donanemab confirms that Aβ clearance can indeed slow clinical decline in biomarker‐verified early AD patients [9, 10, 117]. However, moderate therapeutic magnitude, inherent ARIA risk, high treatment cost, long‐term infusion burden, and strict enrollment criteria limit their widespread clinical application [7, 8, 9, 10, 118]. More importantly, Aβ is more likely an early initiating trigger of AD pathogenesis. Once tau spreading, glial neuroinflammation, synaptic loss, and irreversible neuronal death are established, isolated Aβ clearance cannot fully halt or reverse disease progression. Therefore, future anti‐Aβ therapy should be integrated into an early, precision‐based combined intervention framework, rather than regarded as an ultimate standalone therapeutic solution. Defining the optimal therapeutic time window, identifying most responsive subpopulations, and rationally combining anti‐Aβ regimens with anti‐tau, anti‐inflammatory, metabolic modulatory, and nonpharmacological interventions will constitute the core research priorities in the next stage of AD translational medicine.

### 3.4 Tau‐Targeted DMTs

NFTs formed by abnormal phosphorylation of tau protein constitute another core pathological hallmark of AD, which is directly correlated with neuronal loss and cognitive decline. Accordingly, increasing efforts have been devoted to developing interventions that inhibit tau aggregation or block interneuronal propagation of pathological tau to slow disease progression. Tau‐targeted DMTs include first‐generation anti‐tau monoclonal antibodies (e.g., gosuranemab, tilavonemab, semorinemab), second‐generation anti‐tau monoclonal antibodies (e.g., BMS‐986446, bepranemab, E2814), and tau‐targeting antisense oligonucleotide (ASO) such as diranersen.

First‐generation anti‐tau monoclonal antibodies predominantly target the N‐terminal fragment of tau, aiming to block interneuronal transmission of pathological tau species. Phase 2 trials of gosuranemab, tilavonemab, and semorinemab demonstrated no significant cognitive or functional improvements, despite reduced levels of free tau in CSF [166, 167, 168]. Systematic reviews confirmed that these agents exhibited comparable efficacy to placebo, with favorable safety profiles [11]. These findings indicate that clearance of extracellular free tau alone is insufficient to modify disease progression.

Given the lack of clinical efficacy with early tau antibodies, second‐generation agents have shifted focus toward epitopes within the aggregation‐prone, seeding‐competent microtubule‐binding region (MTBR). Blocking this domain is hypothesized to more effectively inhibit the propagation of tau pathology compared with N‐terminal targeting. Multiple second‐generation anti‐tau antibodies have completed Phase 1 studies and advanced to Phase 2 evaluation.

For instance, Phase 1 results of BMS‐986446 (PRX005) revealed that intravenous administration achieved CNS penetration of 0.2% relative to plasma concentrations, with favorable safety and tolerability. A Phase 2 trial (NCT06268886) is ongoing in early AD patients to assess changes in cognitive and functional outcomes, with completion expected in 2027 [121].

The Phase 2 trial of bepranemab (UCB0107) for MCI or mild AD dementia was completed in May 2024 (NCT04867616). Although the study failed to meet its primary endpoint measured by CDR‐SB, subgroup analysis demonstrated clinical benefits in patients with low baseline tau levels and noncarriers of APOE4. In this subgroup, bepranemab slowed CDR‐SB deterioration by one‐third and reduced decline in the secondary endpoint ADAS‐Cog by half. Tau‐PET imaging confirmed a 58% reduction in tau deposition rate compared with placebo. The agent exhibited a favorable safety profile without evidence of ARIA [122].

E2814 (etalanetug) is currently being evaluated in Phase 2/3 trials (NCT06602258, NCT05269394) to assess its efficacy as monotherapy or in combination with lecanemab on tau propagation, cognitive and functional outcomes, amyloid pathology, and CSF neurofilament light (NfL) chain levels in MCI or mild dementia patients. Completion is expected in 2028 [123, 124].

Nevertheless, development of second‐generation anti‐tau antibodies has encountered setbacks. For example, posdinemab reduced CSF free and total phosphorylated Tau217 levels but failed to slow cognitive decline relative to placebo, leading to termination of development [169]. BIIB076 demonstrated target engagement, with a 50% reduction in CSF mid‐region‐bearing tau content 1 week postinfusion. However, adverse events at the highest dose led to premature trial discontinuation [170].

Additionally, diranersen is an ASO targeting MAP tau (MAPT) mRNA, which selectively reduces MAPT premessenger RNA levels to inhibit de novo synthesis of all tau isoforms, including posttranslationally modified and toxic tau species. Phase 1b results demonstrated that intrathecal diranersen significantly reduced regional tau levels measured by PET in mild AD patients. In the long‐term open‐label extension, CSF total tau and phosphorylated tau181 exhibited dose‐dependent and sustained reductions [171]. Although the Phase 2 trial did not meet its primary dose‐response endpoint, clinical decline was attenuated across all dose groups, accompanied by significant reductions in CSF tau levels and brain tau pathology. Notably, the lowest‐dose group (60 mg) showed the strongest responses across cognitive and clinical endpoints, with declines reduced by 42% on the ADAS‐Cog13, 50% on the MMSE, and 26% on the CDR‐SB [172].

Compared with Aβ, tau pathology exhibits a stronger correlation with cognitive decline, neuronal loss, and disease severity, underscoring the theoretical significance of tau‐targeted therapy. However, the lack of clear clinical benefits with multiple first‐generation anti‐tau antibodies highlights unique challenges in tau‐directed interventions [166, 167, 168]. First, tau is predominantly intracellular, limiting antibody accessibility to pathological tau species. Second, distinct tau conformations, PTMs, and disease stages may correspond to divergent toxic mechanisms.

Future tau‐targeted strategies should evolve from simple free tau clearance toward precise identification and blockade of propagation‐competent tau seeds, pathological MTBR conformations, or upstream MAPT expression. The emergence of ASO‐based nucleic acid therapeutics provides a novel approach to reduce total tau levels; however, careful evaluation is warranted regarding long‐term safety, delivery routes, and potential impacts on physiological tau functions [125, 171].

### 3.5 Active Immunization Vaccines

Active immunization vaccines are designed to induce long‐lasting endogenous antibody production, thereby avoiding repeated intravenous infusion of exogenous monoclonal antibodies. The development of AD vaccines has evolved from first‐generation full‐length Aβ vaccines to second‐generation Aβ vaccines optimized through epitope refinement and immune bias regulation, as well as novel tau vaccines targeting phosphorylated tau or the tau MTBR.

AN1792 was the first Aβ vaccine to enter clinical trials. Long‐term follow‐up demonstrated reduced cerebral Aβ plaque burden and evident plaque clearance upon postmortem examination. Nevertheless, patients still progressed to end‐stage severe dementia. Critically, several participants developed meningoencephalitis, leading to early trial termination [173, 174]. Consequently, subsequent AD vaccine design has prioritized avoiding detrimental T‑cell immune responses. Second‐generation Aβ vaccines include CAD106, UB‐311 and ABvac40. CAD106 utilizes fragmented Aβ peptides retaining only B‐cell epitopes to minimize unwanted T‑cell activation. Its Phase 2b trial confirmed robust serum antibody induction without CNS inflammation. Asymptomatic ARIA occurred in six strong immune responders. Although definite clinical efficacy was not established, the trial verified that active Aβ vaccination can safely induce sustained antibody responses in AD patients [12]. UB‐311 consists of two synthetic peptides targeting the Aβ1–14 B‐cell epitope and employs a Th2‐biased delivery platform. Early studies reported a 100% immune response rate among 19 patients with mild‐to‐moderate AD, with local injection‐site swelling and agitation as the most common adverse events [126]. Its Phase 2a trial further validated overall safety and durable immunogenicity in mild AD [127]. ABvac40 targets the C‐terminus of Aβ40. Phase 1 results showed overall adverse event rates comparable to placebo, with no detected ARIA‐E, ARIA‐H or cerebral microbleeds. Approximately 92% of recipients generated specific anti‐Aβ40 antibodies after three injections, indicating favorable safety, tolerability, and consistent immunogenicity [175]. Given the stronger correlation of tau pathology with neuronal loss and clinical disease progression, active tau immunization represents another viable therapeutic direction. Leading tau vaccine candidates in clinical development include AADvac1, ACI‐35.030, and JACI‐35.054. AADvac1 targets the MTBR of tau. Phase 2 data confirmed satisfactory safety, good tolerability, and robust IgG antibody induction in mild AD patients [128]. Posthoc analyses revealed that AADvac1 reduced the accumulation rate of NfL chain by 56% and glial fibrillary acidic protein (GFAP) by 73% in plasma p‑Tau217‑positive individuals, suggesting potential modulation of neurodegenerative and neuroinflammatory fluid biomarkers. However, no statistically significant improvements were observed in clinical endpoints including CDR‐SB and ADCS‐ADL‐MCI‐18, necessitating larger trials for validation [129].

Both ACI‐35.030 and JACI‐35.054 are vaccines targeting C‐terminal phosphorylated tau. A recently published Phase 1b/2a trial in 2025 enrolled 57 early AD participants to compare the safety and immunogenicity of the two formulations. Both vaccines were well tolerated, with injection‐site reactions and headache as the most frequent adverse events and no severe MRI abnormalities. In terms of immunogenicity, ACI‐35.030 induced anti‑p‑Tau IgG in all recipients after a single dose, with further elevation following booster immunization. In contrast, immune responses to JACI‐35.054 were more heterogeneous and required multiple administrations. Notably, significant differences in plasma p‑Tau217 and brain‐derived tau levels were observed in the 900 and 1800 µg ACI‐35.030 dose groups [130].

Current evidence indicates that second‐generation Aβ vaccines including CAD106, UB‐311, and ABvac40 exhibit acceptable safety and immunogenicity, yet consistent clinical efficacy remains lacking. Tau vaccines such as AADvac1 and ACI‐35.030 demonstrate favorable tolerability and preliminary modulation of neurodegenerative and tau‐associated fluid biomarkers. Active vaccination represents a key direction for long‐term intervention and preventive therapy in AD. Compared with passive monoclonal antibody infusion, active immunization offers advantages including lower administration frequency, reduced potential cost, and durable endogenous antibody induction, making it particularly suitable for preventive strategies in high‐risk populations or very early disease stages [12, 126, 127, 128, 129, 175]. Nevertheless, the failure of AN1792 highlights the necessity of balancing robust immunogenicity with safety to avoid harmful T‑cell activation and central inflammation [173, 174]. At present, second‐generation Aβ and tau vaccines display satisfactory safety and immunogenicity but insufficient improvements in clinical endpoints. Future vaccine research should continue biomarker‐stratified trials in earlier disease stages, with long‐term follow‐up to confirm sustained effects on pathological progression and cognitive decline.

### 3.6 Immunomodulatory Therapy

Neuroinflammation is widely recognized as one of the pivotal pathogenic drivers of AD initiation and progression; however, traditional broad‐spectrum anti‐inflammatory approaches have failed to yield consistent clinical benefits. For instance, nonsteroidal anti‐inflammatory drugs including naproxen and celecoxib showed no cognitive benefits in AD clinical trials [176, 177]. Accordingly, contemporary clinical research has shifted toward precise immunomodulatory strategies, including mast cell/microglia modulation, TREM2 pathway targeting, and Treg immune reconstitution.

Masitinib, an oral tyrosine kinase inhibitor, primarily modulates activated neuroimmune cells such as mast cells and microglia [13]. A randomized, placebo‐controlled Phase 3 study published in 2023 enrolled patients with mild‐to‐moderate AD. At Week 24, significant benefits were observed in the primary endpoints ADAS‐Cog and ADCS‐ADL. Masitinib yielded a between‐group difference of −2.15 in ADAS‐Cog (97.5% CI: −3.48 to −0.81, *p* < 0.001) and 1.82 in ADCS‐ADL (97.5% CI: −0.15 to 3.79, *p* = 0.038) versus placebo [131]. Nonetheless, the trial lacked fluid and imaging biomarker evidence to support genuine disease‐modifying effects, warranting further validation. Regardless, masitinib provides proof‐of‐concept support for neuroimmune cell‐targeted AD therapy. Its clinical application is constrained by safety concerns, with common adverse events including neutropenia, pneumonia, hypoalbuminemia, and Stevens–Johnson syndrome [131]. Subsequent studies must further balance therapeutic efficacy and long‐term tolerability.

TREM2 agonists represent one of the most representative precise microglia‐targeted immunomodulatory strategies and are currently undergoing target engagement validation in the CNS. AL002, a humanized monoclonal antibody targeting TREM2, demonstrated acceptable safety and feasible central target engagement in Phase 1. Dose‐dependent reductions in soluble TREM2 were detected in CSF, accompanied by elevated levels of SPP1, sCSF1R, and IL1RN [178], confirming central activation of TREM2‐related signaling. However, the Phase 2 trial in early AD did not show clinical benefits, and fluid biomarkers and amyloid PET imaging also did not show differences, so further research and development were stopped [132].

Treg immune reconstitution has emerged as an innovative clinical direction in recent years, with low‐dose IL‐2 at the forefront of this field. Low‐dose IL‐2 has been shown to expand peripheral Tregs and suppress proinflammatory monocyte activation and circulating inflammatory mediators in AD patients [179]. A recently published Phase 2a trial enrolled 38 individuals with mild‐to‐moderate AD to compare safety, clinical outcomes, and biomarker changes among low‐dose IL‐2 administered every 4 weeks, every 2 weeks, and placebo. All participants completed the trial without serious adverse events or mortality. The 4‐week dosing regimen exerted clearer immunological effects, sustaining Treg expansion and enhancing immunosuppressive function. This regimen also effectively reduced plasma inflammatory mediators including CCL2, CCL11, and IL‐15, while elevating anti‐inflammatory IL‐4 and CCL13 levels. Additionally, CSF Aβ42 levels were significantly improved relative to placebo (*p* = 0.045) [180]. This landmark study advances anti‐AD strategy from simple inflammation suppression toward immune homeostasis reconstruction. Its Phase 2 study is still ongoing, and clear clinical benefits are expected [133].

Overall, immunomodulatory therapy for AD has entered an accelerated clinical validation phase. Current evidence is dominated by early exploratory findings and partial positive signals, requiring larger‐scale, inflammation‐subtype‐stratified RCT to confirm efficacy and suitable patient populations. A major challenge is that neuroinflammation in AD is not uniformly detrimental but exhibits distinct stage dependence and functional heterogeneity. Future immunotherapy should avoid simply copying peripheral anti‐inflammatory paradigms and instead focus on remodeling central immune homeostasis. An optimal immunomodulatory framework should enhance glial phagocytosis, and metabolic adaptability in early disease stages, while restraining chronic inflammation, complement‐mediated synaptic loss, and excessive inflammasome activation during disease progression. Integrating single‐cell omics, spatial transcriptomics, and cerebrospinal/blood inflammatory biomarkers to stratify individual immune states will facilitate precise patient selection and personalized immunomodulatory treatment for AD.

### 3.7 Repurposing of Antidiabetic Agents

Mounting evidence suggests that AD is tightly linked to cerebral insulin signaling dysfunction and abnormal glucose metabolism. Accordingly, antidiabetic drugs have emerged as promising repurposed candidates for symptomatic intervention. Clinical research focuses not merely on hypoglycemic efficacy, but on whether these agents can ameliorate cerebral energy metabolism, synaptic function, neuroinflammation, and neurodegenerative progression. Three major research strategies have been established: GLP‐1 receptor agonists, intranasal insulin, and insulin sensitizers.

GLP‐1 receptor agonists (GLP‐1RAs) represent one of the most attractive research directions, with liraglutide and semaglutide as representative agents. Preclinical studies have demonstrated that GLP‐1RAs ameliorate cognitive impairment and glucose metabolic disorders in AD animal models, exerting anti‐inflammatory and antioxidant effects [181, 182, 183, 184]. In recent years, clinical exploration of GLP‐1RAs for AD has gradually advanced. The ELAD Phase 2b trial published in 2025 evaluated the efficacy and safety of liraglutide in mild‐to‐moderate AD. A total of 204 nondiabetic patients with mild‐to‐moderate AD received liraglutide or placebo for 52 weeks. The primary endpoint, changes in cerebral glucose metabolic rate, did not reach statistical significance. Nevertheless, the secondary endpoint of the AD Assessment Scale‐Executive Domain (ADAS‐Exec) favored liraglutide over placebo (0.15; 95% CI: 0.03–0.28; unadjusted *p* = 0.01), with satisfactory overall safety and tolerability [14]. Furthermore, semaglutide, a drug that was once highly anticipated in the field of AD, did not demonstrate clinical benefit. Both the EVOKE and EVOKE+ Phase 3 trials were negative, with no significant differences observed in the primary endpoint, CDR‐SB, or in secondary endpoints, including ADCS‐ADL‐MCI, ADAS‐Cog‐13, MoCA, AD Composite Score, MMSE, and composite *Z* score (based on the three outcome measures CDR‐SB, ADCS‐ADL‐MCI, and ADAS‐Cog‐13 from baseline to Week 104) [134].

Intranasal insulin enhances central insulin signaling via the nose‐to‐brain pathway, while avoiding peripheral adverse reactions and hypoglycemia risk. A landmark Phase 2/3 multicenter clinical trial published in 2020 enrolled patients with MCI and AD dementia to assess the feasibility, safety, and efficacy of intranasal insulin. The treatment was generally safe and well tolerated, yet no definite and stable positive outcomes were observed in primary cognitive and functional endpoints [135]. However, secondary analyses revealed encouraging signals: 12‐month intranasal insulin slowed the progression of deep and frontal white matter hyperintensity volume, accompanied by improvements in CSF biomarkers including Aβ42, Aβ42/Aβ40, Aβ42/T‐tau, as well as subtle cognitive benefits [136]. A recent Phase 2A/B randomized trial further indicated that intranasal insulin modulates cerebral blood flow (CBF) and fractional anisotropy FA, and reduces plasma GFAP levels. It also regulates inflammatory biomarkers in CSF, such as IFN‐γ, eotaxin, and IL‐6, implying neuroprotection potentially mediated by immune modulation [137]. These findings suggest that intranasal insulin may interfere with cerebral leukoencephalopathy and AD‐related pathological processes, though definitive clinical benefits remain unconfirmed. In addition, therapeutic efficacy is highly dependent on the reliability of nasal delivery devices, calling for further optimization of intranasal administration systems [135].

Research on insulin sensitizers for AD is mainly represented by metformin and pioglitazone. Metformin is one of the most widely investigated repurposed drugs, yet available evidence is mostly derived from small‐sample exploratory studies and observational data. A 2017 randomized, double‐blind, placebo‐controlled crossover pilot study recruited 20 nondiabetic individuals with MCI or mild AD, receiving metformin and placebo alternately for 8 weeks each. The results indicated that metformin was associated with improved executive function, and posthoc analysis revealed increased cerebral perfusion in the middle orbitofrontal cortex after metformin intervention [138]. Nevertheless, the small sample size and short treatment duration preclude definitive conclusions regarding efficacy. Large‐scale randomized trials of metformin targeting aMCI are still ongoing, focusing on whether it delays cognitive decline, with additional assessments of cortical thickness, white matter hyperintensities, plasma biomarkers, and cerebral Aβ/tau burden (NCT04098666) [185]. Despite low current evidence level, metformin remains a promising candidate for AD repurposing.

In contrast, large‐scale Phase 3 preventive trials of pioglitazone have yielded negative results. The TOMMORROW study enrolled 3494 cognitively normal participants stratified by high or low dementia risk, aiming to explore whether low‐dose pioglitazone delays the onset of MCI due to AD. The results showed that pioglitazone failed to significantly prevent the occurrence of cognitive impairment [186]. This represents one of the most robust negative findings in this field, suggesting that simple intervention targeting the PPAR‐γ/insulin sensitivity pathway hardly achieves successful primary or secondary prevention of AD. Based on current clinical evidence, pioglitazone is not recommended for routine prevention or treatment of AD.

## 4 Advances in Nonpharmacological Therapies for AD

Given the complexity of clinical manifestations and pathological mechanisms in AD, single‐dimensional intervention is often unsatisfactory, making comprehensive multimodal management a clinical consensus. Current pharmacological treatments exert limited effects on delaying disease progression and ameliorating core symptoms. Accordingly, nonpharmacological therapies have emerged as vital complementary strategies with increasingly prominent clinical value. Accumulating studies have confirmed that nonpharmacological interventions not only improve cognitive function, but also relieve behavioral and psychological symptoms of dementia, maintain ADL, and elevate quality of life (QoL). Diversified approaches including exercise therapy, physical neuromodulation, sensory stimulation, and cognitive‐psychological rehabilitation are reshaping the comprehensive management model of AD, offering new therapeutic options for patients with poor drug response or intolerance to medication side effects (Figure 5).

**FIGURE 5:**
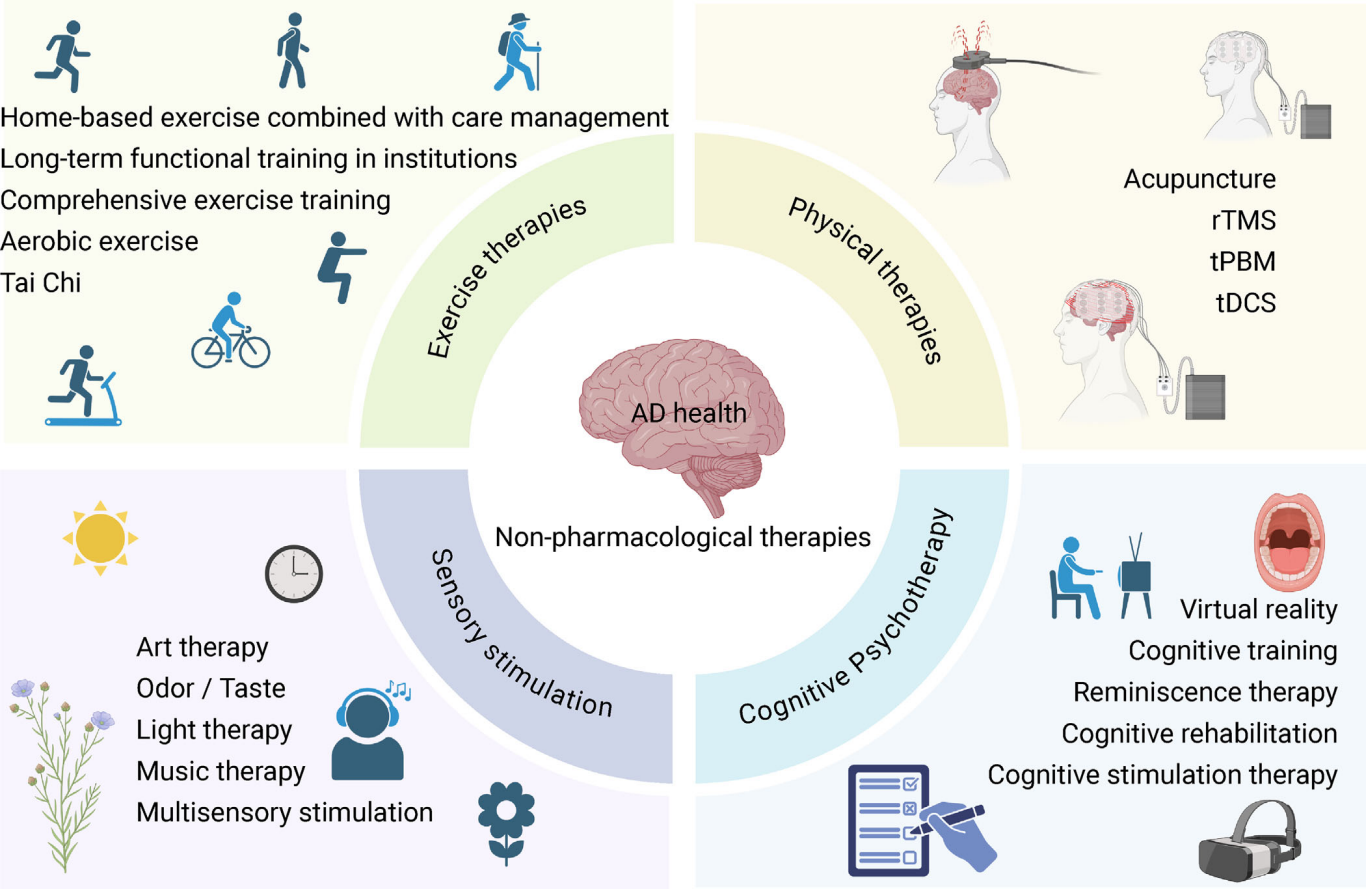
Nonpharmacological therapy for Alzheimer's disease. Nonpharmacological interventions have shown promising prospects in alleviating behavioral and psychological symptoms, improving activities of daily living, and enhancing the quality of life in Alzheimer's disease (AD) patients. Common nonpharmacological therapies for AD include exercise therapies, physical therapies, sensory stimulation therapies, and cognitive psychotherapy. *Abbreviations*: rTMS, repetitive transcranial magnetic stimulation; tDCS, transcranial direct current stimulation; tPBM, transcranial photobiomodulation.

### 4.1 Exercise Therapy

Common exercise interventions for AD include aerobic exercise, combined comprehensive training, home‐based exercise combined with caregiver management, and long‐term functional training in institutional settings [187, 188]. Existing evidence generally indicates that exercise cannot reverse established AD pathological progression; nevertheless, as a nonpharmacological adjuvant therapy, it can moderately slow the decline of daily living ability, improve physical fitness and balance performance, and exert favorable effects on neuropsychiatric symptoms and cognitive outcomes in certain patients [189].

The landmark FINGER study published in The Lancet demonstrated that multidomain intervention combining dietary adjustment, physical exercise, cognitive training (CT) and vascular risk factor monitoring could preserve or improve cognitive function in high‐risk elderly populations [190]. In recent years, multiple clinical trials have explored the cognitive benefits of exercise. For instance, a recent three‐arm RCT evaluated the effects of moderate‐intensity aerobic exercise and resistance training in older adults with MCI. Six months of aerobic exercise exerted positive impacts on global cognition, while resistance training specifically improved executive function. However, only 93 participants completed the trial without biomarker support, limiting its value as merely preliminary exploratory evidence [191].

Mechanistic studies have further elucidated the underlying effects of exercise therapy. Moderate‐intensity aerobic exercise slows the atrophy of hippocampal volume and temporal cortical thinning and attenuates the progression of cerebral white matter hyperintensities. Such beneficial effects are relatively modest and tend to reverse after intervention withdrawal [192]. Notably, neither high‐intensity nor moderate‐intensity exercise alters AD‐related gene expression profiles [193]. Traditional Chinese Tai Chi has been proven to improve prefrontal hemodynamics and enhance executive function and reaction speed [194], as well as ameliorate static and dynamic balance, cardiopulmonary fitness, and overall QoL [195].

In addition, recent systematic reviews and meta‐analyses have concluded that the stable benefits of exercise intervention in AD are mainly reflected in physical endurance, functional independence, and neuropsychiatric symptom relief, whereas the improvement in global cognition is relatively limited and greatly affected by exercise intensity, frequency, duration, and patient adherence [187, 196, 197]. Therefore, exercise therapy is currently more appropriate as an important supportive strategy in the comprehensive management of AD, rather than an independent curative intervention.

### 4.2 Physical Therapy

Physical therapy for AD mainly encompasses repetitive transcranial magnetic stimulation (rTMS), transcranial direct current stimulation (tDCS), transcranial photobiomodulation (tPBM), and other noninvasive neuromodulation techniques. Cumulative evidence indicates that physical interventions delay functional decline by improving cognitive and motor performance and may directly modulate cognition‐related brain regions via enhancing cerebral blood perfusion, regulating cortical excitability, and reshaping neural network connectivity.

RCTs have demonstrated that rTMS targeting the precuneus improves global cognition, ADL, and psychiatric symptoms in patients with mild‐to‐moderate dementia [198]. Combined rTMS applied to the left dorsal lateral prefrontal cortex and lateral temporal lobe not only ameliorates cognitive function and daily living ability but also markedly enhances effective connectivity within brain networks [199]. Meta‐analyses have confirmed that as an adjuvant strategy, rTMS exerts prominent superimposed benefits when combined with CT, Tai Chi, or tDCS. Such multimodal protocols effectively elevate global cognitive performance in AD and MCI, with therapeutic benefits sustained for a period after treatment cessation [200].

tDCS has been shown to improve verbal ability and memory in the short term, although long‐term persistent effects remain controversial [201, 202]. Multisite anodal tDCS combined with cognitive stimulation can slow cognitive decline more significantly than sham stimulation, modulate cortical electrical activity, and enhance coherence of high‑ and low‑frequency electroencephalography oscillations. These neurophysiological alterations are positively correlated with cognitive improvement [203].

As an emerging noninvasive brain stimulation technique, tPBM has attracted increasing attention for its potential to improve cognition in elderly populations. Systematic reviews indicate that tPBM at specific wavelengths (800–1064 nm) ameliorates brain function by modulating mitochondrial activity, restoring cytochrome *C* oxidase activity and ATP production, and exerting anti‐inflammatory and immunomodulatory effects [204]. Particularly in older women, a high‐risk group for AD, tPBM serves as a safe and feasible intervention, with potential benefits in alleviating cognitive impairment, regulating cerebral hemodynamics, and conferring neuroprotection [205].

Beyond electromagnetic neuromodulation, traditional physical and natural therapies also play a complementary role in AD management. Warming needle moxibustion, integrating acupuncture with thermal stimulation of moxa, yields superior outcomes compared with acupuncture alone or routine medication. It significantly elevates MMSE scores, reduces CDR grades, and improves ADL in AD patients [206]. Given the critical involvement hypoxia in AD pathogenesis, oxygen therapy exhibits promising neuroprotective potential. Hypoxia is closely linked to abnormal amyloid metabolism, tau hyperphosphorylation, and oxidative stress in AD. Oxygen therapy may retard disease progression and relieve clinical symptoms by improving cerebral blood volume, mitochondrial function, and neurotrophic factor levels [207].

Systematic reviews and meta‐analyses further highlight substantial heterogeneity across existing studies in stimulation parameters, treatment courses, and outcome measures, limiting the comparability and generalizability of current evidence [208] Collectively, physical therapy is recognized as a valuable supplementary component in the integrated care framework of AD, with core advantages in slowing functional deterioration, alleviating partial cognitive symptoms, and promoting QoL. Large‑sample RCTs with standardized protocols are still needed to clarify long‐term efficacy and optimal intervention parameters.

### 4.3 Sensory Stimulation Therapy

Sensory stimulation therapy represents a supportive intervention framework centered on regulating sensory input and environmental experience. Common modalities include olfactory/gustatory stimulation, music therapy, art therapy, light therapy, and multisensory stimulation (MSS/Snoezelen). Current evidence suggests that these approaches are best applied as nonpharmacological adjuvant interventions, with primary benefits in relieving behavioral and psychological symptoms, improving sleep, and enhancing QoL. Improvements in global cognitive function are generally modest and less durable.

Although self‐consciousness is impaired but not completely lost in AD patients, emotion‐based sensory stimulation provides a key pathway to reawaken self‐identity. Emotion serves as a core component of personal identity construction. Evoking emotionally laden autobiographical memories via olfactory, gustatory, or musical cues can temporarily enhance memory capacity, emotional stability, and self‐awareness in AD patients [209]. Familiar odors and tastes can directly retrieve deep emotion‐associated memories, helping rebuild self‐continuity and improving QoL for both patients and caregivers [210].

Music therapy is widely applied owing to its favorable effects on cognition, emotion, and behavior in AD. Mechanistic reviews indicate that musical intervention modulates CBF, regulates neurotransmitter transmission (including GABAergic and monoaminergic systems), remodels synaptic plasticity, and modulates hormonal release to optimize brain function [211]. Systematic evaluations of clinical trials confirm that music therapy improves cognitive outcomes compared with standard care, with more pronounced benefits observed during active musical participation and creation [212]. Beyond cognitive enhancement, music therapy exerts broad beneficial effects, alleviating anxiety, depression, agitation, sleep disturbance, and physical pain while elevating overall QoL [213].

Art therapy shows unique advantages in managing apathy, a prevalent neuropsychiatric symptom in early AD. A RCT comparing painting/coloring activities with cognitive arithmetic training revealed that both interventions partially preserve cognitive function, whereas art therapy achieved superior improvements in apathy rating and QoL [214]. Museum‐based art appreciation and creation programs also enhance subjective well‐being, emotional status, and life quality in AD patients [215]. Recent systematic reviews further demonstrated that artistic interventions significantly ameliorate global cognition, neuropsychiatric symptoms, and depressive mood. These findings suggest that compared with pure CT, artistic activities emphasizing motivation, emotional expression, and psychological satisfaction are more targeted for alleviating specific behavioral and psychological symptoms.

Light therapy is one of the most widely adopted nonpharmacological interventions for AD. Evidence supports its benefits in alleviating depression, apathy, agitation and affective disturbance, as well as regulating circadian rhythm and ameliorating sleep disorders. Bright light therapy effectively corrects circadian misalignment, sleep‐onset insomnia, nocturnal awakening, insufficient total sleep duration, and poor sleep architecture, thereby optimizing circadian rhythmicity [216]. Accordingly, bright light therapy is a reliable nonpharmacological strategy for managing sleep‐related abnormalities in AD.

Snoezelen therapy, a classic comprehensive multisensory environmental intervention, delivers soothing modulation through multidimensional stimulation including soft light, sound, tactile, and olfactory cues. Existing systematic reviews indicate that MSS can relieve agitation, apathy, depression, and anxiety in AD patients. Nevertheless, overall evidence quality remains limited, with high heterogeneity in intervention protocols, duration, and enrolled populations, precluding consistent and definitive conclusions on efficacy [217].

In summary, the clinical value of sensory stimulation therapy lies mainly in optimizing symptom management and care quality. Further large‐scale standardized RCTs are required to clarify long‐term efficacy, optimal stimulation parameters, and suitable patient subgroups.

### 4.4 Cognitive and Psychological Interventions

Cognitive and psychological interventions represent a patient‐centered therapeutic system that focuses on functional maintenance and psychosocial adaptation. Major modalities for AD include reminiscence therapy (RT), cognitive stimulation therapy (CST), CT, and cognitive rehabilitation (CR). Such interventions can partially preserve or improve global cognition, verbal communication, subjective QoL, and goal‐oriented ADL.

RT is a classic psychosocial intervention designed to stimulate memory through structured recall of past life experiences. Systematic reviews have demonstrated that individual or group‐based RT, delivered at least once weekly for approximately 12 weeks, effectively improves cognitive performance, QoL, and neuropsychiatric symptoms in elderly AD patients [218, 219]. Individualized RT yields greater benefits in cognitive function and emotional regulation, whereas group‐based RT better enhances interpersonal communication and social interaction [220, 221], making it a highly recommendable nonpharmacological strategy in long‐term care settings.

CST delivers general cognitive skill training through enjoyable social activities [222]. Classic RCTs have verified its favorable effects on cognitive performance and QoL [223]. Systematic reviews further confirmed that CST stably improves cognition, physical and mental well‐being, communication skills, and social engagement in dementia patients [224]. A recent single‑blind pragmatic RCT indicated that CST also enhances caregiver–patient relationship quality and caregivers’ own QoL [225].

In contrast, CT emphasizes repetitive practice targeting specific cognitive domains such as memory, attention, and executive function. RCT have shown that CT can slow cognitive decline and promote initiative, episodic memory, and emotional stability [226]. Nevertheless, systematic reviews highlight substantial heterogeneity across studies in training content, frequency, duration, and outcome measures, limiting the consistency of therapeutic effects [227].

CR adopts a goal‐oriented framework, implementing compensatory and adaptive training centered on personalized real‐life objectives to improve daily functional capacity. Randomized trials have validated the definite value of CR in improving goal‐related daily activities, occupational performance, and QoL [228, 229]. Neuroimaging studies suggest that CR may reshape memory‐related neural activation patterns; however, benefits tend to be domain‐specific and do not generalize to all cognitive subdomains [228].

With technological advances, cognitive psychological interventions are undergoing digital transformation. Beyond conventional multimodal cognitive approaches, virtual reality (VR) has opened new avenues for cognitive assessment and training. VR provides an ecologically valid cognitive evaluation environment and enables visuospatial reasoning training within three‐dimensional immersive scenarios. This novel interactive paradigm enhances cognitive engagement and exhibits great potential for alleviating depression and other neurocognitive symptoms [230, 231].

The value of nonpharmacological therapies should not be regarded merely as an adjunct to medication, but as an integral component of long‐term comprehensive AD management. Especially in the preclinical and MCI stages, nonpharmacological interventions are optimally combined with biomarker screening, risk factor control, and early pharmacological treatment to establish a low‐risk, sustainable preventive framework. Current nonpharmacological protocols remain highly heterogeneous across studies, hindering direct comparison and clinical generalization. Future research should establish standardized intervention regimens, objective outcome indicators, and long‐term follow‐up systems to standardize this field and elevate the overall evidence level of nonpharmacological therapy.

## 5 Application of Nanoscale Drug Delivery Systems

The BBB severely restricts the penetration of most small‐molecule drugs and nearly all macromolecular biologics into the CNS, resulting in extremely low cerebral bioavailability of conventional therapeutics [232]. Nanoscale drug delivery systems not only enhance BBB permeability but also provide an engineered, combinatorial, and targeted therapeutic platform for the complex multifactorial pathology of AD. By modulating particle size, surface charge, hydrophilicity/hydrophobicity, ligand modification, and stimuli‑responsive properties, nanocarriers simultaneously achieve drug protection, targeted brain delivery, sustained release, and multitarget synergistic intervention [17, 233].

In preclinical AD models, multiple nanoplatforms—including liposomes, polymeric nanoparticles, solid lipid nanoparticles (SLNs), dendrimers, cell membrane‑biomimetic nanoparticles, metal‐based nanoparticles, and carbon‐based nanoparticles—have demonstrated promising capacity to facilitate BBB penetration, elevate intracerebral drug accumulation, and reduce systemic toxicity [234]. Moreover, nanotherapies targeting core pathological hallmarks, including Aβ aggregation, tau hyperphosphorylation, neuroimmune microenvironment dysregulation, and oxidative stress, have become a major research hotspot. In particular, nucleic acid nanomedicines combined with gene‐editing technologies offer a highly promising strategy for precision targeted therapy of AD (Figure 6).

**FIGURE 6:**
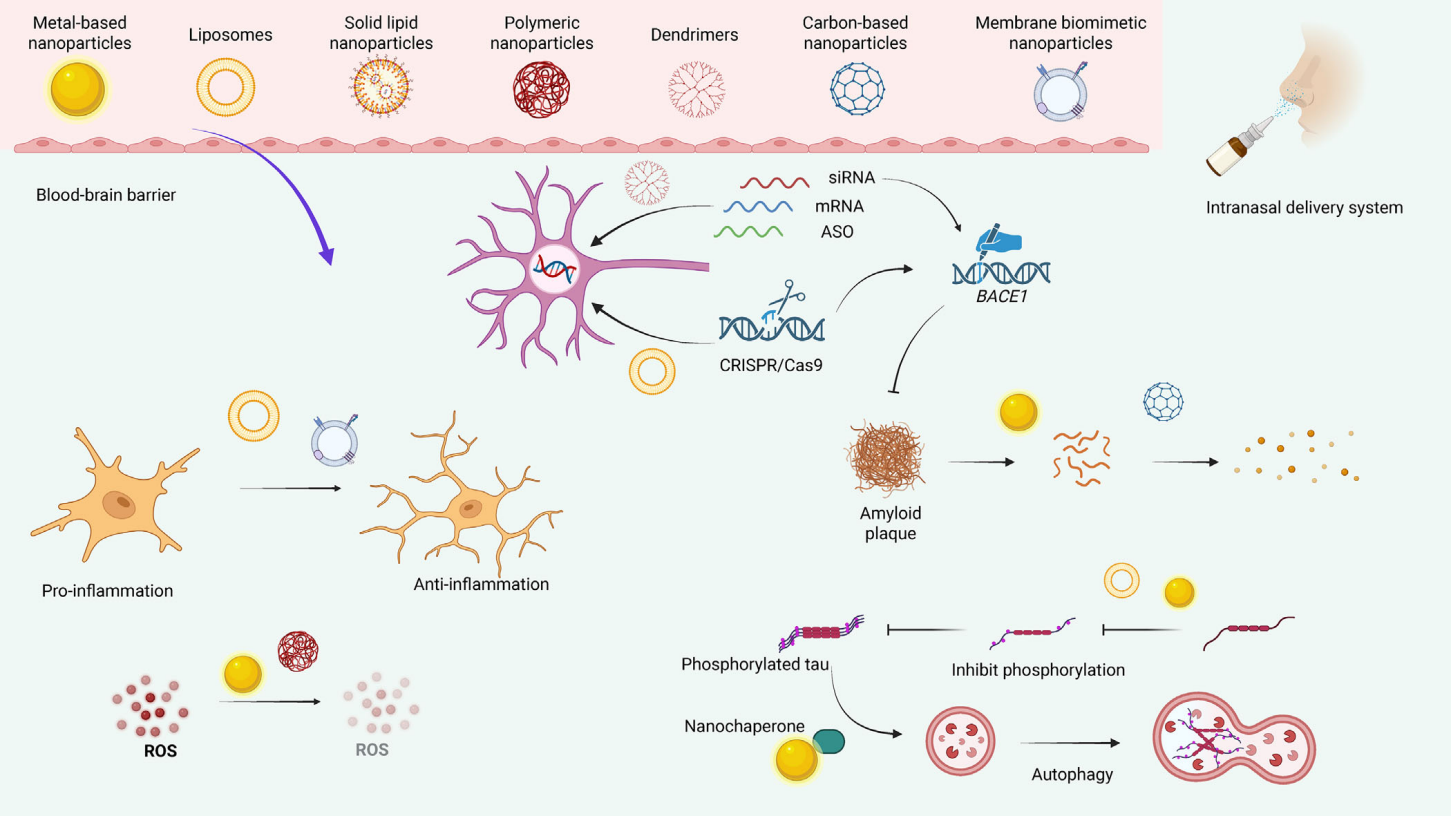
Nanodrug delivery systems for Alzheimer's disease. Nanodrug delivery systems not only enhance the blood–brain barrier penetration efficiency of drugs, but also provide an engineered, combinatorial, and targeted therapeutic platform for the complex pathology of AD. Liposomes, polymeric nanoparticles, solid lipid nanoparticles, dendrimers, membrane biomimetic nanoparticles, metal‐based nanoparticles, and carbon‐based nanoparticles all exhibit considerable therapeutic potential. In addition, nucleic acid nanomedicines combined with gene editing technologies offer a highly promising strategy for targeted therapy of AD. *Abbreviations*: ASO, antisense oligonucleotide; ROS, reactive oxygen species.

### 5.1 Nanoplatform Strategies for BBB Penetration

Conventional small‐molecule drugs suffer from limited brain penetration, while macromolecular agents such as antibodies, proteins, siRNA, mRNA, and CRISPR‐related components can hardly cross the BBB via natural diffusion. Nanocarriers can efficiently enhance brain delivery through multiple pathways, including RMT, adsorption‐mediated transcytosis, cell membrane biomimetic homing, focused ultrasound‐induced reversible BBB opening, and the nose‐to‐brain route [235]. Among these, RMT represents the most mature and widely adopted strategy for brain‐targeted nanocarrier design. Representative RMT targets include transferrin (Tf) receptor (TfR), low‐density LRP1, insulin receptor, and lactoferrin receptor. Surface modification with ligands such as Angiopep‐2, ApoE‐mimetic peptides, Tf, lactoferrin, and RVG29 peptide can strengthen the binding and transcytosis of nanoparticles across brain endothelial cells [236, 237].

Research on TfR‐targeted delivery has evolved from direct Tf conjugation toward more biologically stable and low‐competition strategies based on monoclonal antibodies and short peptides [238, 239]. Traditional Tf‐modified nanoparticles often exhibit poor transcytosis efficiency due to competitive binding by abundant endogenous circulating Tf [240]. To overcome this limitation, antibodies (e.g., 8D3) and their fragments targeting noncompetitive TfR epitopes have been developed. For instance, bispecific antibodies and the Brain Shuttle technology enable efficient endocytosis and transmembrane transport without interfering with endogenous iron metabolism [238, 241]. In addition, affinity peptides such as RVG29 and T7 possess low immunogenicity, small molecular weight, and easy surface functionalization; when decorated on lipid nanoparticles (LNPs), they markedly facilitate targeted mRNA delivery into the brain [239].

For LRP1 targeting, ApoE‐modified nanoparticles have demonstrated prominent advantages in enhancing brain delivery. LNPs adsorb endogenous ApoE and achieve bidirectional BBB penetration via LRP1‐mediated transcytosis [242]. Polymeric vesicles designed based on multivalent interaction not only promote drug entry into the brain but also modulate LRP1 transport polarity to facilitate peripheral efflux and clearance of cerebral Aβ, achieving dual therapeutic benefits [243]. As a peptide specifically binding to LRP1, Angiopep‐2 has been extensively used to functionalize diverse nanocarriers such as dendrimers and LNPs for high‐efficiency brain‐targeted delivery [244, 245].

Notably, excessive ligand affinity or over‐dense surface decoration does not always improve delivery; overly strong binding may trap nanocarriers within endothelial cells or trigger lysosomal degradation, thereby reducing parenchymal release efficiency. Future design should precisely balance receptor affinity, intracellular trafficking routing, brain parenchymal release, and off‐target peripheral distribution [246].

Beyond active ligand targeting, the protein corona effect has emerged as an innovative brain delivery strategy. Peptide‐functionalized gold nanoparticles (AuNPs) can regulate the composition of blood‐derived protein coronas. Preformation of ApoE‐enriched protein coronas significantly enhances BBB permeability without altering nanoparticle surface chemistry, offering a practical route for brain targeting [247]. Furthermore, cell membrane biomimetic camouflage using erythrocyte membranes, microglial membranes, or brain metastatic tumor cell membranes enables nanoparticles to evade immune clearance and cross the BBB via inherent cellular homing mechanisms [248, 249].

Physical auxiliary approaches also facilitate nanomedicine brain delivery. Photothermal effects can transiently open the BBB, concurrently promoting drug penetration and enabling synergistic photothermal ablation of amyloid plaques [250]. Focused ultrasound combined with microbubbles achieves localized, reversible BBB opening. When integrated with piezoelectric nanomaterials, this strategy even permits wireless neuromodulation and on‐demand drug release in deep brain regions [251, 252]. For example, Piezo1‐targeted magnetic nanobubbles generate mechanical and electrical signals under transcranial magnetoacoustic stimulation, simultaneously promoting BBB opening and activating microglial Aβ clearance [253].

The nose‐to‐brain pathway provides a noninvasive shortcut bypassing the BBB directly into the CNS. Lactoferrin‐functionalized LNPs delivered intranasally efficiently transported BACE1 siRNA and α‐mangostin for synergistic AD therapy via olfactory and trigeminal nerve pathways [254]. Thermosensitive in situ gels incorporated with nanostructured lipid carriers prolong nasal residence time and markedly enhance brain absorption and neuroprotective effects of drugs such as felodipine and quercetin [255, 256]. This noninvasive route greatly improves patient compliance and represents a key developmental direction for future AD drug delivery.

Nevertheless, BBB penetration rate cannot be simply equated to therapeutic efficacy. BBB integrity dynamically changes across AD stages: the barrier remains relatively intact in early AD, whereas middle‐to‐late disease is characterized by increased permeability, pericyte degeneration, altered endothelial transporter expression, and NVU dysfunction [104]. Consequently, the same nanocarrier may exhibit distinct brain distribution and safety profiles at different disease stages. For RMT‐based strategies, targets including TfR, insulin receptor, and LRP1 are susceptible to receptor saturation, peripheral off‐target expression, and disease‐related expression fluctuation, compromising delivery stability [18, 257]. Future nanodelivery research should incorporate AD staging, BBB integrity evaluation, and brain target expression profiling to optimize delivery performance.

### 5.2 Nanoplatforms Targeting Aβ Aggregation

Aβ plaques constitute the most prominent pathological hallmark of AD and serve as a core therapeutic target for nanomedicine development. Current nanotherapeutic strategies mainly focus on inhibiting Aβ aggregation, disassembling mature amyloid fibrils, and promoting Aβ clearance. A broad spectrum of nanomaterials, including AuNPs, selenium nanoparticles, graphene quantum dots (GQDs), carbon dots, cerium‐based nanoparticles, and peptide self‐assembled nanosystems, can interfere with Aβ aggregation kinetics through electrostatic interaction, hydrophobic interaction, π–π stacking, and β‐sheet structural disruption [234].

In recent years, various functionalized nanomaterials have exhibited remarkable antiamyloidogenic capacity. AuNPs are widely investigated owing to facile surface modification. AuNPs decorated with β‐sheet‐disrupting peptides can specifically recognize and bind Aβ aggregates, blocking amyloid fibrillation [247]. Chiral nanomaterials also display unique advantages; chiral Fe_7_Se_8_ nanorods not only inhibit Aβ aggregation but also remodel the neuroimmune microenvironment via modulating the gut–brain axis [258]. Carbon‐based nanomaterials such as GQDs and carbon dots interfere with Aβ self‐assembly via π–π stacking and hydrophobic forces. Nitrogen‐doped GQDs exert stronger antiamyloid activity than undoped counterparts [259]. Ampholytic carbon dots simultaneously inhibit Aβ fibrillation, disassemble mature fibrils, and reduce amyloid‐induced cytotoxicity [260]. In addition, short‐peptide assemblies with soft‐aggregation behavior bind Aβ via hydrophobic interaction, effectively suppressing and reversing amyloid fibrillation [261].

Combining photothermal therapy with nanoprobes represents another innovative strategy against Aβ pathology. Well‐designed near‐infrared responsive nanoprobes can specifically target Aβ plaques. Upon light irradiation, they generate localized hyperthermia or ROS to physically disrupt or oxidatively degrade Aβ aggregates, while sparing normal surrounding tissues [262, 263, 264]. For instance, red‐emitting cerium‐based carbon dots integrate the anti‐inflammatory properties of curcumin and the redox activity of cerium ions, enabling simultaneous fluorescent Aβ imaging and efficient photothermal disaggregation of amyloid fibrils [265].

Recent advances in intranasal targeted LNPs further reinforce the dual therapeutic paradigm of Aβ clearance plus pathological pathway modulation. For example, dual‐targeted nanoparticles intranasally delivering BACE1 siRNA and rapamycin simultaneously suppress Aβ production and enhance autophagy‐mediated clearance, ultimately improving cognitive performance in AD model animals. Intranasal delivery bypasses the BBB and achieves high brain exposure via olfactory and trigeminal nerve pathways [266]. Latest studies further engineered targeted LNPs as a two‐pronged β‐amyloid nanoscavenger for nose‐to‐brain delivery, highlighting that nanocarriers function not only as simple delivery vehicles but also as intrinsic therapeutic agents capable of direct Aβ capture, aggregation inhibition, and intracerebral clearance via surface functionalization [254].

### 5.3 Nanoplatform Strategies Targeting Tau Pathology

Unlike extracellular Aβ, Tau protein is predominantly localized within neurons. Accordingly, Tau‐targeted nanotherapeutics must not only cross the BBB but also achieve neuronal internalization, endosomal escape, cytosolic release, and selective recognition of pathologically folded Tau conformers. Current Tau‐directed nanoplatforms mainly focus on inhibiting Tau aggregation, suppressing PTM, recognizing pathological tau species, and facilitating their clearance [267, 268].

In terms of small‐molecule delivery, methylene blue (MB) is one of the most extensively investigated tau aggregation inhibitors. Nevertheless, clinical translation is hindered by insufficient brain delivery, complex mechanisms, and off‐target effects. To enhance brain targeting, Liu et al. constructed black phosphorus nanosheets loaded with MB, further integrated into a thermosensitive intranasal hydrogel system. Intranasal administration bypasses the BBB, while the hydrogel prolongs nasal mucosal retention and enables sustained drug release. In vivo studies demonstrated prolonged cerebral drug release, inhibition of tau neuropathology, restoration of mitochondrial function, mitigation of neuroinflammation, and improved cognitive performance in AD mice [269]. Similarly, green‐synthesized oral silver nanoparticles coloaded with MB and Moringa oleifera reduced astrogliosis and proinflammatory cytokines, enhanced autophagy and antioxidant capacity, and suppressed tau hyperphosphorylation by inhibiting GSK‑3β activity in tau pathology models [270]. Targeting the same GSK‑3β pathway, SLNs loaded with indirubin derivatives efficiently penetrated the BBB and downregulated tau phosphorylation [271]. Cell membrane camouflage has also been applied for tau targeting; melanoma membrane‐camouflaged selenium nanoassemblies cross the BBB and specifically target intracellular phosphorylated tau, alleviating pathological progression via antioxidant and anti‐inflammatory mechanisms [272]. Collectively, nanodelivery systems not only overcome poor brain penetration of tau inhibitors but also exert multipathway synergistic effects through antiaggregation, antioxidation, anti‐inflammation, and autophagy modulation.

Beyond conventional small‐molecule delivery, the nanochaperone strategy has emerged as an innovative direction for intervening intracellular tau pathology. Since physiological tau maintains microtubule stability and axonal transport, ideal therapies should avoid nonselective depletion of normal tau. Biomimetic molecular chaperone‐based nanomaterials selectively bind misfolded or aggregation‐prone tau, block further fibrillization, and promote clearance via the autophagy–lysosome pathway. Recent studies have shown that customized nanochaperones selectively sequester pathological tau and activate in situ autophagy to isolate toxic species, preserving tau homeostasis and microtubule integrity [273]. This represents an evolutionary shift from simple aggregation inhibition toward precise recognition, sequestration, and targeted clearance of pathological tau.

Although Aβ‐ and tau‐targeted nanodelivery strategies possess solid pathological rationales, their clinical translation requires rational evaluation. Nanocarriers improve drug stability, prolong circulation time, and enhance brain targeting, thereby elevating central bioavailability of anti‐Aβ and anti‐tau agents [234, 269]. However, Aβ and tau pathological burdens vary substantially across disease stages, brain regions, and individual patients. Future protein‐targeted nanotherapies should adopt precise stratification based on Aβ/tau biomarkers, PET imaging, and disease progression. Particularly in advanced stages with widespread tau propagation, massive synaptic loss, and persistent neuroinflammation, enhancing isolated anti‐Aβ delivery is unlikely to reverse cognitive decline, rendering multitarget codelivery more therapeutically meaningful.

### 5.4 Nanoplatforms for Regulating the Neuroinflammatory Microenvironment

Neuroinflammation acts as a core amplifier of AD progression. Microglia, the resident immune cells in the brain, serve as a pivotal therapeutic target via phenotypic polarization from proinflammatory M1 toward anti‐inflammatory M2 and intermediate regulatory states. In early AD, microglia exert homeostatic functions by phagocytosing Aβ; however, prolonged exposure to Aβ, tau, and DAMPs drives microglia toward a proinflammatory, phagocytic‐dysfunctional, or disease‐associated microglial phenotype. Nanodelivery systems can reshape the cerebral immune microenvironment by targeting microglial receptors, modulating miRNA/mRNA profiles, delivering anti‐inflammatory drugs, or functioning as nanozymes [274].

In situ microglia reprogramming expands nanocarriers from simple nucleic acid delivery vehicles toward functional engineering tools for immune cells, representing a cutting‐edge frontier in AD nanomedicine. For example, engineered microglia‐targeted LNPs deliver synthetic phagocytic receptor mRNA to microglia, enhancing Aβ phagocytosis and degradation without overt proinflammatory activation [275, 276]. Mannose‐modified LNPs delivering anti‐miR‑17 to microglia ameliorated AD pathological and behavioral deficits, confirming that miRNA modulation is a viable entry point for neuroimmune intervention [277]. Such studies highlight that future AD nanotherapies should extend beyond single Aβ/tau targeting to integrate regulation of the microglia–astrocyte–neuron network.

Furthermore, cell membrane camouflage endows synthetic nanoparticles with inherent microglia‐targeting bioactivity. Nanoparticles cloaked with neutrophil, erythrocyte, platelet, or stem cell membranes exhibit prolonged circulation and inflammatory tropism [278]. Specifically engineered membrane coatings, such as neural stem cell membranes overexpressing the Gas6 receptor, enable precise microglial targeting and enhanced Aβ clearance [279]. In another design, liposomes camouflaged with apoptotic bodies derived from brain metastatic tumor cells codeliver manganese dioxide nanozymes and rapamycin. By mimicking physiological efferocytosis, they induce microglial polarization toward an anti‐inflammatory phenotype and effectively resolve chronic neuroinflammation [249]. To suppress excessive complement activation, C1q‐targeted microglial membrane‐camouflaged lipid carriers loaded with alectinib successfully inhibit complement‐mediated synaptic phagocytosis and neuroinflammatory responses [248].

### 5.5 Antioxidative Stress and Mitochondria‐Targeted Nanoplatforms

Oxidative stress and mitochondrial dysfunction occur throughout AD pathogenesis. Aβ and tau pathologies induce mitochondrial membrane potential collapse, impaired electron transport chain function, excessive ROS generation, and neuronal apoptosis. Conversely, accumulated ROS further accelerates Aβ aggregation, tau hyperphosphorylation, and proinflammatory cytokine release, forming a self‐perpetuating vicious cycle. Consequently, nanozymes with SOD‐, CAT‐, GPx‐, and peroxidase‐mimicking activity, as well as intrinsically antioxidant nanomaterials, have attracted intensive research attention [280]. In particular, mitochondria‐targeted nanozymes equipped with mitochondrial targeting moieties achieve precise subcellular localization, directly preserving mitochondrial membrane potential, reducing lipid peroxidation, and inhibiting neuronal apoptosis [281].

Metal‐based nanozymes such as Au@Cu_2_Se heterogeneous trimers exhibit dual GPx and SOD‐like activity, efficiently eliminating excessive mitochondrial ROS, restoring membrane potential, and protecting neurons from oxidative damage [282]. Selenium‐enriched nanomaterials also serve as potent antioxidants. Baicalin‐ and myricetin‐decorated selenium nanoparticles (BMSe@BSA) exert multitarget effects by inhibiting amyloid formation and downregulating proinflammatory cytokine expression [283]. Natural antioxidants such as curcumin suffer from poor bioavailability; encapsulation within PLGA nanoparticles markedly enhances brain delivery, antioxidant and anti‐inflammatory efficacy, and improves cognitive performance in AD animal models [284].

Overall, anti‐inflammation, antioxidation, and mitochondrial protection constitute critical application directions for AD nanotherapy, targeting common downstream injury pathways independent of Aβ/tau pathology. Chronic neuroinflammation, ROS accumulation, mitochondrial energy metabolic failure, and metal ion imbalance mutually reinforce one another, driving persistent neuronal damage. Anti‐inflammatory and antioxidant nanosystems therefore serve as valuable microenvironment modulators in combinatorial therapeutic regimens. Notably, inflammation and oxidative stress are not entirely detrimental: moderate early microglial activation facilitates Aβ clearance, while excessive immune suppression may impair intrinsic cerebral clearance capacity. Future nanodelivery systems should emphasize lesion microenvironment responsiveness and controlled on‐demand release to avoid disrupting immune homeostasis in normal brain tissue.

### 5.6 Nanodelivery Strategies for Gene Editing

With advancing understanding of AD susceptibility genes, gene therapy has emerged as a promising disease‐modifying modality. Therapeutic nucleic acids including siRNA, miRNA inhibitors, mRNA, ASOs, and CRISPR/Cas systems can fundamentally regulate pathological protein production and neuroimmune homeostasis. Nevertheless, clinical translation is severely limited by inherent vulnerability of nucleic acids to nuclease degradation and poor BBB penetration. Nanocarriers provide effective biological protection, stable circulation, and targeted brain delivery for these macromolecular therapeutics [285].

RNA interference is widely employed to silence BACE1, the key enzyme driving Aβ generation. For instance, RVG29–peptide‐modified dendrimers and α‐mangostin‐functionalized LNPs efficiently cross the BBB and penetrate brain parenchyma, enabling potent BACE1 silencing, reduced Aβ deposition, and rescued memory deficits [254, 286]. Multifunctional calcium phosphate nanocores codelivering BACE1 siRNA and rutin achieve synergistic therapeutic effects combining gene silencing, anti‐inflammation, and antioxidation [287]. Furthermore, engineered stem cell‐derived exosomes intranasally codeliver BACE1 siRNA and berberine, ameliorating cognitive impairment and facilitating neural repair in AD models. This highlights exosomes as versatile platforms integrating nucleic acid protection, brain targeting, and intrinsic bioactivity [288]. Nanodelivery of nucleic acids also opens new avenues for tau‐targeted therapy. BBB‐permeable LNPs enable intravenous mRNA delivery into the CNS, achieving robust transgene expression in neurons and astrocytes and providing a universal platform for tau‐related mRNA therapeutics [289]. Tripartite motif‐containing protein 11 (TRIM11) is an ATP‐independent disaggregase capable of dissolving pathological tau fibrils and promoting proteasomal clearance. Recent studies demonstrated that BBB‐penetrating LNPs delivering TRIM11 mRNA achieve widespread brain transgene expression in 3×Tg‐AD mice, preferentially enriching in neurons, facilitating clearance of hyperphosphorylated tau aggregates, and modulating neuroinflammation [290]. Nanodelivered mRNA encoding endogenous tau‐clearing factors therefore represents an innovative paradigm for early disease‐modifying intervention in AD. Of particular interest, CRISPR/Cas9 gene editing offers the potential for long‐term therapeutic benefits in AD. LNPs enabling nonviral delivery of CRISPR/Cas9 components allow precise editing or knockout of disease‐associated genes. For example, LNP‐delivered CRISPR activation upregulates metallothionein 3 expression in astrocytes, markedly enhancing Aβ endocytosis and clearance [291]. Among nonviral vectors, third‐generation PAMAM dendrimers and novel ionizable lipid‐based LNPs effectively protect nucleic acid cargos and facilitate high transfection efficiency in neural cells [292, 293]. Additionally, emerging epigenetic editing strategies utilizing nanocarriers to deliver epigenetic modifying enzymes enable fine‐tuned regulation of AD‐related gene expression without altering genomic DNA sequences [294].

Collectively, nucleic acid nanodelivery stands among the most cutting‐edge frontiers in AD nanomedicine. Advances in ASOs, gene silencing, miRNA modulation, and inflammatory pathway intervention hinge on safe, stable, cell‐selective delivery of nucleic acids to neurons, microglia, or astrocytes. Nanocarriers shield nucleic acids from enzymatic degradation and enhance brain targeting and cellular internalization via surface functionalization. However, narrow therapeutic windows, off‐target effects, innate immune activation, long‐term gene regulation, and cell‐type selectivity require rigorous evaluation. Future development must prioritize safety upon repeated administration and potential impacts on physiological APP/tau metabolism, immune homeostasis, and neural plasticity.

## 6 Conclusion and Prospects

The pathogenesis of AD cannot be fully explained by isolated Aβ deposition or tau NFTs; instead, it arises from long‐term interplay and cascade amplification of multiple interconnected pathological processes. Cumulative evidence reviewed herein demonstrates that pathological proteins including Aβ, tau, and TDP‐43 mutually interact through structural polymorphism, PTM, LLPS, cell‐to‐cell propagation, and impaired protein degradation pathways, collectively forming the core basis of proteostasis dysfunction in AD [26, 27, 28, 29, 30, 31, 32]. On this foundation, phenotypic transformation of microglia and astrocytes, aberrant activation of the NLRP3 inflammasome and TREM2 signaling, complement‐mediated synaptic loss, mitochondrial dysfunction, lipid overload, cerebral insulin resistance, and disrupted metal ion homeostasis further expand localized protein pathology into widespread network‐level brain injury [21, 56, 61, 65, 70, 71, 79, 83, 87, 88]. Accordingly, AD should be conceptualized as a chronic neurodegenerative disorder driven by multiprotein, multicellular, and multisystem dysregulation, rather than a linear disease governed by a single pathological cascade.

Mechanistically, AD is characterized by robust positive feedback loops across distinct pathological modules. Abnormal Aβ and tau aggregation trigger mitochondrial damage, calcium dyshomeostasis, and oxidative stress. Mitochondrial dysfunction further amplifies neuroinflammation via excessive ROS release, mtDNA leakage, and cGAS–STING pathway activation [67, 68, 69]. Lipid metabolic disturbance and APOE4‐related cholesterol trafficking deficits impair synaptic repair and Aβ clearance, while driving proinflammatory microglial phenotypic transition via intracellular lipid accumulation [22, 79, 80, 81, 82]. Gut and oral microbiota dysbiosis disrupt BBB integrity and activate central immune responses through circulating LPS, depleted short‐chain fatty acids, periodontal pathogen virulence factors, and peripheral inflammatory signaling [97, 98, 99, 100, 101]. Progressive BBB deterioration and glymphatic system dysfunction further impede drainage of Aβ, tau, and metabolic waste, establishing a self‐sustaining vicious cycle among toxic protein deposition, vascular impairment, and chronic neuroinflammation [104, 110, 111]. Such networked pathology underscores the need for future research to prioritize temporal trajectories and crosstalk among interconnected mechanisms.

Therapeutically, AD management is undergoing a paradigm shift from symptomatic relief toward disease modification. ChEIs and memantine remain cornerstone clinical regimens, maintaining practical value in preserving cognition, managing behavioral symptoms, and sustaining ADL across mild‐to‐moderate and moderate‐to‐severe AD [139, 140, 145, 146, 147]. The advent of anti‐Aβ monoclonal antibodies marks a milestone in DMT. Clinical trials of lecanemab and donanemab confirm that targeted Aβ clearance can partially slow cognitive and functional decline in biomarker‐verified early AD patients [9, 10, 117]. Nonetheless, limitations remain, including modest clinical magnitude, ARIA risks, mandatory MRI surveillance, elevated susceptibility in APOE4 carriers, high treatment costs, and restricted accessibility [7, 8, 9, 117, 118]. Anti‐Aβ immunotherapy therefore represents not a definitive solution, but rather the beginning of an era embracing precise stratification and combinatorial intervention.

Although many natural compounds exert potent anti‑inflammatory and antioxidant effects in preclinical studies, several representative candidates such as resveratrol have failed to yield satisfactory cognitive benefits in clinical trials. Such unsatisfactory translational outcomes can be attributed to insufficient blood–brain barrier permeability, low oral bioavailability, unsuitable dosage regimens, disease heterogeneity among AD patients, and late intervention at advanced pathological stages. Future therapeutic development should center on early detection, biological phenotyping, stage‐specific intervention, and multimodal combination therapy. In preclinical and MCI stages, Aβ clearance, metabolic modulation, sleep optimization, and lifestyle intervention offer optimal therapeutic windows. In advanced phases characterized by robust tau propagation and overt neuroinflammation, combinatorial strategies targeting tau pathology, immune regulation, mitochondrial protection, and neural network repair become indispensable. Future clinical trials should stratify participants based on Aβ‐PET, tau‐PET, cerebrospinal and blood biomarkers, genetic background, inflammatory profiles, metabolic phenotypes, and vascular risk burden, rather than relying solely on clinical symptomatic diagnosis. Novel strategies including tau‐targeted therapeutics, active vaccination, TREM2/NLRP3 immunomodulation, GLP‐1 receptor agonists, and oral small‐molecule anti‐Aβ agents require rigorous validation in biologically defined populations, optimal disease stages, and long‐term follow‐up cohorts [11, 12, 13, 14, 15, 126].

Furthermore, the role of nonpharmacological therapy and long‐term integrated care should be further elevated within the AD prevention and management framework. Although exercise, physical neuromodulation, sensory stimulation, sleep intervention, CT, and social engagement rarely induce rapid alterations in pathological imaging endpoints comparable to pharmacological agents, they exhibit superior safety, long‐term feasibility, and cumulative benefits by improving cerebral perfusion, synaptic plasticity, metabolic homeostasis, sleep architecture, inflammatory tone, and glymphatic clearance. Future AD care models should evolve from monotherapy toward multidimensional comprehensive intervention, shifting from singular pathological protein clearance toward whole‐course management integrating cognitive function, daily competence, behavioral and psychological symptoms, caregiver burden, and QoL.

Notably, nanoscale drug delivery systems provide a transformative technological breakthrough for AD treatment. Beyond merely facilitating BBB penetration, nanoplatforms enable customizable functional design tailored to distinct pathological modules. Engineered nanocarriers can be rationally constructed for pathological protein recognition, aggregation inhibition, and clearance promotion targeting Aβ and tau [234, 267, 268, 273]; for targeted delivery of anti‐inflammatory, antioxidant, and mitochondria‐protective agents against neuroimmune and oxidative injury [274, 280, 281]; and for stable delivery of siRNA, miRNA, mRNA, CRISPR/Cas9, and other nucleic acid therapeutics to rectify gene and RNA regulatory dysfunction [285]. This offers a versatile platform enabling multitarget, combinatorial, and stage‐stratified AD therapy.

Nevertheless, substantial challenges remain before widespread clinical translation of nanomedicines. First, conventional AD animal models cannot fully recapitulate the chronic progressive course, regional pathological heterogeneity, and complex comorbidities in elderly human patients. Second, long‐term biosafety concerns including nanomaterial accumulation, immune activation, complement reaction, hepatic and splenic clearance, renal excretion, and potential neurotoxicity require systematic long‐term evaluation. In addition, the large‐scale production of complex nanoformulations faces challenges related to manufacturing processes, particle size distribution, drug‐loading capacity, release profiles, batch‐to‐batch consistency, and quality control, while their complex pharmacokinetic and biodistribution profiles further complicate regulatory evaluation. Therefore, future research should move beyond the pursuit of BBB penetration efficiency alone and systematically evaluate effective drug exposure, lesion‐targeting selectivity, long‐term safety, scalable production, and clinical manufacturability, while further establishing standardized quality‐control and regulatory evaluation frameworks [18, 235]. Addressing these critical issues will be essential for translating nanodelivery systems from promising technological platforms into clinically applicable tools for AD treatment.

## Author Contributions

Gaoshuang Fu conceptualized the manuscript and drafted the initial manuscript. Mingmin Pan prepared/improved the figures. Qingling Sun drafted the initial manuscript. Guangxin Yue and Tong Lei offered direction and guidance and provided relevant resources. All authors revised the manuscript and approved the final manuscript.

## Funding

We are grateful for the fund support of Fundamental Research Funds for the Central public welfare research institutes (ZZ18‐YQ‐060, CYZ‐202514) and Scientific and Technological Innovation Project of China Academy of Chinese Medical Sciences (CI2026A01314).

## Ethics Statement

The authors have nothing to report.

## Conflicts of Interest

The authors declare no conflicts of interest.

## Data Availability

The authors have nothing to report.
